# Targeting Oxidative Stress in Acute Pancreatitis: A Critical Review of Antioxidant Strategies

**DOI:** 10.3390/nu17152390

**Published:** 2025-07-22

**Authors:** Laura Ioana Coman, Daniel Vasile Balaban, Bogdan Florin Dumbravă, Horia Păunescu, Ruxandra-Cristina Marin, Mihnea Costescu, Lorena Dima, Mariana Jinga, Oana Andreia Coman

**Affiliations:** 1 Medical Center for Diagnosis, Outpatient Treatment and Preventive Medicine, 011794 Bucharest, Romania; laura.coman21@yahoo.com; 2 Faculty of Medicine, Internal Medicine and Gastroenterology Discipline, “Carol Davila” University of Medicine and Pharmacy, 020021 Bucharest, Romania; vbalaban@yahoo.com (D.V.B.); mariana.jinga@umfcd.ro (M.J.); 3 Gastroenterology Department, “Dr. Carol Davila” Central Military University Emergency Hospital, 010825 Bucharest, Romania; 4 Department of Gastroenterology, “Prof. Dr Dimitrie Gerota” Emergency Hospital, 030167 Bucharest, Romania; bogdan.dumbrava92@yahoo.ro; 5 Discipline of Pharmacology, Clinical Pharmacology and Pharmacotherapy, “Carol Davila” University of Medicine and Pharmacy, 020021 Bucharest, Romania; mihnea.costescu@umfcd.ro (M.C.); oana.coman@umfcd.ro (O.A.C.); 6 Faculty of Medicine, Transilvania University of Brasov, 500017 Brasov, Romania; lorena.dima@unitbv.ro

**Keywords:** acute pancreatitis, oxidative stress, inflammation, antioxidant, anti-inflammatory

## Abstract

Acute pancreatitis (AP) is among the most frequent gastroenterology emergencies, with hospital admission rates on the rise in recent decades. However, a specific treatment for this condition is still lacking. Mitochondrial damage induced by oxidative stress is regarded as the key event in the pathophysiology and initiation of cellular damage in AP. In the early stages of AP, the oxidant–antioxidant balance changes rapidly, and there are significant data regarding the reduced serum levels of antioxidants, with this event being correlated with the clinical severity of pancreatitis. Therefore, addressing oxidative stress could represent a potential therapeutic target in AP. In this comprehensive review, we aimed to provide an update on current evidence regarding clinical and experimental data on antioxidant use in AP, focusing on human studies investigating the effects of single and combined antioxidant supplementation. Although a multitude of animal studies demonstrated that antioxidant therapy has beneficial effects in experimental AP by reducing oxidative injury, inflammatory markers, and ameliorating histological outcomes, human trials showed predominantly conflicting results, with some studies suggesting benefit while others showed no effect, or even potential harm, when antioxidants were administered in high doses or in combination. Moreover, some antioxidants with beneficial results in experimental settings did not show the same efficacy when translated to human studies, which may be a consequence of either inappropriate dosage, route of administration and duration of therapy, or altered pharmacodynamics in vivo. In conclusion, oxidative stress plays a key role in the pathophysiology of AP by enhancing acinar cell injury, inflammation, and systemic complications. Future studies should be centered on optimized dosing strategies, early administration protocols, targeted patient selection, and delivery methods of proper pharmaceutical forms.

## 1. Introduction

Acute pancreatitis (AP) is among the most frequent gastroenterology emergencies, with hospital admission rates on the rise in recent decades. Moreover, its burden is further increased by a high readmission rate due to recurrent attacks or complications [1]. Despite its significant morbidity and mortality, the current available treatment is not specific, and no novel agent has been introduced in the therapeutic armamentarium of AP. The lack of specific therapies derives from insufficient knowledge of its pathogenesis.

Among the intimate molecular mechanisms that drive the pancreatitis process, mitochondrial damage by oxidative stress is an important pathogenic pathway in AP, acting as the principal regulator of apoptotic and necrotic cell death. Mitochondrial dysfunction has been reported in both acinar and ductal cells [2] and acts as a key event in the pathophysiology and initiation of cellular damage in AP [3]. In mild pancreatic injury, the loss of mitochondrial membrane integrity triggers the intrinsic (mitochondrial) apoptotic cascade by releasing cytochrome C and activating caspase. In the case of severe oxidative stress or calcium overload, rapid ATP depletion is produced by the opening of the mitochondrial permeability transition pore (MPTP), impaired ion homeostasis, and a loss of energy, signs of necrotic cell death. Mitochondrial impairment can induce either apoptosis or necrosis, depending on the type and severity of acinar cell injury [4,5,6]. In vivo and in vitro studies have indicated that mitochondrial depolarization and ATP loss are highly linked to necrosis. Meanwhile, mitochondrial outer membrane permeabilization is the key point of apoptosis initiation [7,8]. Excessive ROS enhance these mitochondrial effects, further exacerbating pancreatic injury and inflammation.

It is well-recognized that necrosis and cell apoptosis during a flare of AP are driven by the early intrapancreatic activation of zymogens, especially cathepsin and trypsinogen. On the other hand, experimental evidence challenged this dogma and showed that trypsin activation plays a role in early acinar cell damage but not in the subsequent inflammatory response [9].

Alongside necrosis and apoptosis, ferroptosis is a distinct form of regulated caspase-independent cell death precipitated by iron-induced lipid peroxidation [10], which may be important in the context of oxidative stress in AP, especially in hyperlipidemic states [11]. Ferroptosis is characterized by the accumulation of lipid ROS, the depletion of plasma membrane polyunsaturated fatty acids, and the failure of glutathione-dependent detoxification mechanisms, particularly the glutathione peroxidase 4 (GPX4) pathway. Recent studies have shown that ferroptosis is implicated in pancreatic acinar cell death and inflammation in experimental models of AP, thus being a possible target for therapeutic interventions, potentially consisting of iron chelators and lipophilic antioxidants like omega-3 fatty acids, vitamin D, and vitamin E [12].

**Figure 1:**
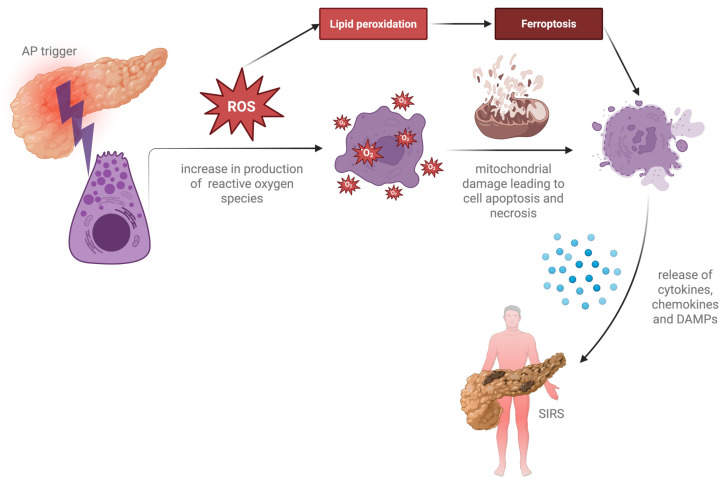
Summary of oxidative stress and antioxidant pathways in acute pancreatitis. Excessive ROS generation induces mitochondrial damage, leading to apoptotic and necrotic cell death. Ferroptosis, an iron-dependent form of regulated necrosis characterized by lipid peroxidation, is also triggered by oxidative stress. Created by Biorender.com. AP = acute pancreatitis; ROS = reactive oxygen species; DAMPs = damage-associated molecular patterns; SIRS = systemic inflammatory response syndrome.

Early acinar cell injury triggers the production of oxygen free radicals, highly reactive species inducing lipid oxidation, the oxidative alteration of proteins, mitochondrial damage, and DNA fragmentation [13]. In addition, injured or dead acinar cells release damage-associated molecular patterns (DAMPs), pro-inflammatory cytokines and chemokines that recruit innate immune cells, thus rapidly initiating an acute sterile inflammatory response [14]. Finally, ongoing inflammation in AP results in tissue injury beyond the pancreas, resulting in systemic inflammatory response syndrome (SIRS) [15] (Figure 1). In the early stages of AP, the oxidant–antioxidant balance changes rapidly, and there are significant data regarding the reduced serum levels of antioxidants in patients with AP, with this event being correlated with the clinical severity of pancreatitis [16,17].

Among many biomarkers of oxidative stress, the malondialdehyde (MDA) plasmatic level, as a marker of lipid peroxidation, was shown to be correlated with the severity of AP at the beginning of the disease [18]. Additionally, superoxide dismutase (SOD), a free oxygen radical scavenger with marked antioxidant activity in vivo, shows decreasing plasmatic levels during AP, and a recent study demonstrated that SOD activity was highly accurate in predicting negative outcomes and mortality in early AP [19]. Moreover, an altered antioxidant status correlates with a higher incidence of complications after an episode of AP, and oxidative stress was shown to be involved in AP progression [13]. A recent study found high levels of oxidative markers in the pancreatic necrotic fluid of patients with walled-off necrosis (WON), with these markers being significantly increased in patients with infected compared to sterile WON [20]. Thus, an infection of pancreatic necrotic fluid may be related to the increased production of reactive oxygen species (ROS), and antioxidant therapy could be beneficial in preventing this complication, which is strongly associated with increased mortality.

Experimental models showed that mitochondrial damage caused by oxidative stress leads to decreased ATP production, reduced intracellular calcium clearance, and finally apoptosis. Addressing the role of mitochondrial dysfunction in the pathogenesis of AP by delivering ATP to protect from cell injury is being researched in trials aiming at restoring energy levels in the pancreas [21]. However, although antioxidants are already successfully used in other acute inflammatory conditions (such as acute ethanolic hepatitis), the current treatment of AP does not include antioxidant supplementation, and their therapeutic role in AP remains controversial.

Although nowadays the pathophysiology of AP is mostly understood, current therapeutic strategies are mainly supportive. Given the multifactorial nature of AP, including biliary, alcohol-related, and metabolic, treatment management is directed to reduce systemic complications and to modulate the inflammatory cascade.

Among the cornerstone strategies, early intensive intravenous fluid resuscitation and timely initiation of enteral feeding were associated with reduced mortality, lower rates of infections in necrotic tissues, and shorter lengths of hospital stay (LoHS) [22,23].

These therapeutic interventions help preserve pancreatic perfusion, prevent gut barrier disorder, and reduce the systemic inflammatory response. Thus, they represent first-line therapy in the acute management of AP, while adjuvant therapies like antioxidants can serve to attenuate oxidative injury and downstream complications. Addressing oxidative stress could thus theoretically represent a potential therapeutic target for AP. In this comprehensive review, we provide an update on current evidence regarding clinical and experimental data on antioxidant use in AP. Furthermore, we aimed to deliver a deeper understanding on the role of antioxidants in AP, with an emphasis on clinical studies as an argument for the real benefit of their supplementation, as well as to clarify the rationale of future research on this topic.

## 2. Materials and Methods

For the purpose of this paper, we searched for data in two databases in September 2024 and on 15 January 2025 for all publications referring to the association between antioxidants and pancreatitis. The search strategy included a PubMed inquiry using the terms—(acute pancreatitis [MeSH Major Topic]) AND (antioxidants [MeSH Major Topic])—which revealed 261 results, and a ScienceDirect search using the combination (“Acute pancreatitis”) and (“Antioxidants”), which, after searching in the Title/Abstract/Keywords of the authors, returned 126 results. We further selected original papers relevant to the study purpose based on title and abstract screening using filters and grouped results for various antioxidant molecules identified throughout the search. In the review-type articles, we also searched inside the bibliography. In-press or online versions of articles found in the last search (15 January 2025) were also included.

## 3. Results

### 3.1. Antioxidants in Monotherapy

#### 3.1.1. Vitamins

1. Alpha-tocopherol

Alpha-tocopherol is a form of vitamin E, along with three other tocopherols and four tocotrienols. Its main role is to act as a fat-soluble antioxidant by scavenging free radicals and protecting cell membranes from ROS. Alpha-tocopherol administration showed significant benefits regarding the improvement in oxidative function and the attenuation of collagen deposition during the development of experimental chronic pancreatitis (CP) in rats [24], as well as increased relative pancreatic weight and improved survival rate [25].

A few experimental studies have suggested that alpha-tocopherol may also have a protective effect in AP, by altering inflammatory gene expression in L-arginine-induced AP in rats [26] and improving pancreatic histopathological scores in cerulein-induced AP [27]. A study comparing the effect of low-dose (200 mg/kg) to high-dose (400 mg/kg) alpha-tocopherol administered intraperitoneally 30 min before the induction of AP with L-arginine in rats on inflammatory markers and histopathologic alterations concluded that high-dose supplementation had a more significant positive impact on inflammation scores and pancreatic edema. However, both groups receiving alpha-tocopherol had a significant overall reduction in AP severity, compared to controls [28].

A small randomized double-blind placebo-controlled trial investigated the effects of alpha-tocopherol supplementation in AP patients, with significant results regarding reduced oxidative stress and inflammation, determined by a significant reduction in MDA, CRP, and IL-6 levels, in parallel with an increased total antioxidant status [29] (Table 1).

Data from experimental models and one small-scale clinical trial suggesting antioxidant and anti-inflammatory effects of alpha-tocopherol in acute pancreatitis look encouraging. Still, the evidence remains preliminary. While findings such as reduced malondialdehyde and cytokine levels are promising, the limited aim and scale of current studies restrict the possibility of the outcomes’ generalization. Comprehensive clinical investigations are needed to validate the therapeutic efficacy of alpha-tocopherol, particularly with standardized dosing protocols and stratification by disease severity.

2. Ascorbic acid

Ascorbic acid (vitamin C) is an important nutrient involved in metabolic processes and antioxidant defense systems. It is a scavenger of ROS and has important roles in many enzymatic reactions as a cofactor: the synthesis of catecholamine, vasopressin, steroids, and carnitine. The dietary intake of vitamin C is sufficient to ensure physiological processes, but in acute and critical illness, the level of ascorbic acid is decreased, together with an increase in the systemic oxidative stress [30].

It has been shown that in AP, plasmatic levels of vitamin C decrease continuously over the first 5 days after admission, and early reduction in serum vitamin C is related to the severity of AP [31].

When the concentrations of vitamin C and its bioavailable form, ascorbic acid, were compared between healthy volunteers and patients with AP, it was found that the patients had very low levels of both. This suggests that the parenteral administration of vitamin C and ascorbic acid could be a potential treatment option for these patients [32].

A systematic review and meta-analysis of nine preclinical and four clinical studies analyzed the effects of ascorbic acid in the treatment of AP, with doses ranging from 500 mg orally to 10 g intravenously administered within 72 to 96 h from disease onset for a duration of 5 days to 2 weeks. Pooled analysis showed a significantly shorter period of hospital stay for patients treated with ascorbic acid, along with reduced pancreatic injury. On the other hand, survival and organ failure rates were not improved [33] (Table 1). Of the four clinical studies included in the meta-analysis, three investigated combined antioxidant therapy (including ascorbic acid), while only one investigated the therapeutic efficacy of high-dose intravenous vitamin C (10 g/day for 5 days) as single therapy on symptoms, complications, hospital stay, and mortality rate in patients with AP. The level of plasma antioxidants (including vitamin C) was significantly decreased in patients with severe AP, while plasma lipid peroxide was significantly increased. Antioxidant levels increased significantly in the treatment group, symptoms resolved, and patients had a higher cure rate (30 vs. 18%), fewer complications, and a shorter hospital stay (9 vs. 13 days) [34] (Table 1).

A case–control study investigated the role of vitamin C in severe AP, concluding that the group receiving high-dose vitamin C had better clinical and biological responses, showing a marked reduction in organ dysfunction, improved tissue perfusion, and a lower mortality rate. Interestingly, they noted a significant decrease in serum vitamin C concentration, which was significantly correlated with the occurrence of pulmonary (*p* = 0.035), renal (*p* = 0.009), and metabolic (*p* = 0.011) dysfunctions in the study group [35] (Table 1).

The therapeutic role of ascorbic acid in AP is controversial. Although several studies and a recent meta-analysis reported improvements in oxidative stress markers and reductions in hospitalization duration, no consistent benefit has been observed in terms of survival or organ failure. These findings underscore the need for more trials to assess its therapeutic potential as an adjuvant in the treatment of AP. Also, future research needs to determine the circumstances in which vitamin C supplementation may be useful, such as the ideal dose, the administration route, and timing in regard to illness onset.

3. Beta-carotene

Beta-carotene, alpha-carotene, and beta-cryptoxanthin are provitamin A carotenoids, which can be converted in vivo to retinol (vitamin A). Beta-carotene is a dietary lipid-soluble antioxidant which can trap free radicals and protect lipids from oxidation [36]. The benefits of beta-carotene administration were studied in many conditions; in AP, the positive effects of beta-carotene were proven in post-ERCP AP and CP [37].

A double-blind trial compared a dose of beta-carotene administered 12 h before ERCP vs. placebo. The incidence of AP was similar in both groups, but patients in the placebo group experienced more severe pancreatitis attacks, which demonstrates some potential protective effects of beta-carotene supplementation in this setting [38] (Table 1).

In addition to antioxidant effects, beta-carotene and its metabolites were shown to regulate pancreatic β-cell function and pancreatic innate immune responses through metabolic and cell signaling processes, with the impact of maintaining glucose homeostasis and potentially preventing diabetes onset [39], which are desirable outcomes in the setting of both recurrent AP and CP.

A recent experimental study found that beta-carotene supplementation in ethanol-induced pancreatic injury in mice showed significant improvement in biochemical and histological parameters [40], with a prior study confirming the antiapoptotic effects of beta-carotene supplementation in chronic ethanol-fed rats [41].

A study assessing the relationship between AP and serum concentrations of alpha-tocopherol, retinol, and beta-carotene found that patients with AP exhibited notably diminished serum levels (*p* < 0.017), with a noteworthy association being observed between the peak CRP value and the lowest serum antioxidant concentrations (*p* < 0.01). In patients with mild AP, the concentrations of retinol and beta-carotene at the final evaluation were significantly greater in comparison to those of patients with severe AP (*p* < 0.05), coinciding with a decrease in CRP concentration [16]. The low serum antioxidant level correlated with the increased severity of the disease may indicate the utility of beta-carotene supplementation in severe AP.

Beta-carotene has demonstrated antioxidant, anti-inflammatory, and metabolic benefits in experimental models and may offer protective effects in AP, showing a potential benefit in post-ERCP settings. Given its low serum levels in severe AP and its possible role in modulating immune and metabolic responses, further clinical studies are required to explore its therapeutic potential in this context.

4. Vitamin D

The term “vitamin D” refers to a class of steroid hormones found in trace levels in food, but which are mostly produced endogenously by the skin’s reaction to ultraviolet (UV) radiation from the sun. Maintaining a healthy mineralized skeleton and controlling calcium homeostasis are the primary roles of vitamin D. Additionally, vitamin D is essential for pleiotropic actions like anti-inflammatory and immune responses, reducing inflammation that can generate oxidative stress, maintaining cell differentiation and proliferation control and inhibiting apoptosis, autophagy, tumor suppression, or fibrosis [42].

Vitamin D influences the expression of certain genes involved in antioxidant defense, promoting the synthesis and action of endogenous antioxidants. Some studies suggest that adequate vitamin D levels are associated with decreased markers of oxidative damage in cells and tissues by reducing oxidative stress [42,43]. Moreover, its active form 1,25-dihydroxycholecalciferol can inhibit iron-dependent lipid peroxidation, thus reducing ferroptosis and protecting cell membranes from ROS-induced damage [44].

Patients with AP are most likely to have vitamin D deficiency or inadequacy, and this has been demonstrated in numerous studies.

From March 2015 to September 2017, Yonsei University Wonju College of Medicine prospectively enrolled patients with AP. At onset, 28.5% of patients had vitamin D insufficiency, while 56.2% had vitamin D deficiency. According to the Atlanta classification, the severity of AP was associated with a higher incidence of vitamin D insufficiency. In terms of predicting severe AP (*p* = 0.015) and ICU admission (*p* = 0.035), vitamin D insufficiency was an independent factor [45].

Using a large retrospective database of 36,087,380 individuals, a study showed that patients with AP had a higher risk of developing vitamin D insufficiency than those without AP (odds ratio [OR]: 1.25, *p* < 0.0001) [46]. In a prospective study on 315 patients, the serum vitamin D level upon admission was found to be inversely correlated with the severity of AP according to the revised Atlanta classification (*p* < 0.001); Ranson and BISAP scores were also statistically significantly related to vitamin D levels, with higher scores in cases of deficient or insufficient levels (<10 ng/mL and 10–19 ng/mL, respectively), but no correlation with ICU admissions or mortality was found [47].

However, there are also studies, particularly case reports on self-medication, which suggest that high doses of vitamin D may induce AP through clinically significant hypercalcemia [48,49].

To date, we have not found any non-clinical or clinical study to investigate whether supplementation with a certain dose of vitamin D has an effect on AP. Vitamin D deficiency or insufficiency may have a negative impact on AP evolution, but there is still no clinical evidence that restoring vitamin D levels before AP onset could lead to better outcomes. Nevertheless, the beneficial effect of vitamin D supplementation was documented to appear due to its antioxidant properties in various pathologies [42,43].

Table 1 summarizes the main characteristics of studies investigating the effect of vitamin supplementation in AP.

**Table 1:** Vitamin supplementation in acute pancreatitis.

Antioxidant	First Author, Year	Study Design and Aim	Dose and Duration of Supplementation	No. of Participants	Outcomes	Adverse Effects
Alpha-tocopherol	Firat et al., 2014 [29]	*Double-blind placebo-controlled RCT.* To investigate the effect of alpha-tocopherol on oxidative stress and inflammation in AP.	400 IU/day orally for 7 days	50	Significantly lower levels of MDA, CRP, and IL-6 (*p* < 0.05); significantly increased total antioxidant status (*p* < 0.05).	None reported ^1^
Ascorbic acid (Vitamin C)	Chooklin et al., 2020 [35]	*Prospective case–control.* To investigate the effect of high-dose ascorbic acid on outcomes in severe necrotizing AP.	4 g/day for 7–8 days	181	Positive impact on organ dysfunction; significantly reduced incidence of infected necrosis (*p* = 0.001) and mortality rate (18.6% vs. 10.6%; *p* = 0.27).	None reported
	Gao et al., 2021 [33] ^2^ studies evaluating combined therapy (ascorbic acid + other antioxidants) were included	*Systematic review and meta-analysis of 4 clinical studies.* To investigate the effect of ascorbic acid on AP outcomes.	Ranging from 500 mg orally to 10 g with IV within 72 to 96 h from AP onset for 5 days to 2 weeks	219	Significantly reduced LoHS (*p* < 0.001); no impact on survival and organ failure.	None reported
	Du et al., 2003 ^2^ [34]	*Prospective randomized case–control.* To investigate the effect of high-dose ascorbic acid on AP.	10 g/day of IV for 5 days in the treatment group vs. 1 g/day of IV for 5 days in controls	84	Significant positive impact on symptom resolution, cure rate (30 vs. 18%), and LoHS (9.34 vs. 13.45 d) (all *p* < 0.05); significant decrease in serum cytokines (*p* < 0.05) and non-significant decrease in CRP; significant improvement in antioxidant status (*p* < 0.05).	None reported
Beta-carotene	Lavy et al., 2004 [38]	*Double-blind placebo-controlled RCT.* To investigate the effect of beta-carotene supplementation in the prevention of post-ERCP AP.	Single-dose 2 g oral beta-carotene 12 h prior to ERCP	321	No impact on the incidence of post-ERCP AP; significant positive impact on the severity of AP—lower incidence of severe AP in the treatment group (*p* < 0.01).	None reported

^1^ no data about adverse effects were mentioned in the study; ^2^ the study was included in the meta-analysis by Gao et al. [33], but reported the supplementation of ascorbic acid as single antioxidant therapy, and therefore, we considered it separately. RCT = randomized controlled trial; AP = acute pancreatitis; MDA = malondialdehyde; CRP = C-reactive protein; IL-6 = interleukin-6; LoHS = length of hospital stay; ERCP = endoscopic retrograde cholangiopancreatography.

#### 3.1.2. Amino Acids and Derivatives

1. L-carnitine

L-carnitine is a vitamin-like amino acid that plays an important role in cellular energy metabolism by transporting fatty acids to mitochondria for beta-oxidation [50]. Another role is to protect tissue from oxidative stress by stabilizing cell membranes, decreasing the production of free radicals, and scavenging hydrogen peroxide [51].

An experimental study investigating the effects of L-carnitine against H_2_O_2_-induced oxidative stress in human hepatocytes demonstrated cytoprotective properties related to the scavenging of ROS, the prevention of lipid peroxidation, and the indirect regulation of fatty acid beta-oxidation through carnitine palmitoyl transferase [52]. The same antioxidant properties of L-carnitine in AP were emphasized in experimental animal studies. Hasan et al. showed that the administration of L-carnitine in combination with meloxicam had benefits by ameliorating the parameters of oxidative stress in induced AP in rats (tumor necrosis factor-α, malondialdehyde, nitric oxide, myeloperoxidase) [53].

A preclinical study showed that the administration of acetyl L-carnitine has protective effects in cerulein-induced AP in rats by modulating oxidative stress through myeloperoxidase and nitric oxide systems [54].

Another study of the therapeutic effect of L-carnitine (500 mg/kg) on L-arginine-induced AP in rats conducted by Soliman et al. highlighted significant biochemical improvement, increased pancreatic tissue glutathione level, and a decrease in the level of inducible nitric oxide synthetase [55].

An animal study aimed to assess the efficacy of acetyl-L-carnitine (ALC) to diminish pain-related behaviors and brain microglial activity along the pain circuitry in cerulein-induced pancreatitis. Pancreatitis was produced with 6-hourly intraperitoneal injections of cerulein (50 µg/kg), 3 days a week for 6 weeks in male C57BL/6J mice. Starting from week 4, mice were given either vehicle or ALC until the experiment’s completion. The results showed that ALC treatment reduced inflammation-induced hypersensitivity, but repeated intraperitoneal injections produced damage to the abdominal wall, which resulted in persistent hypersensitivity [56].

Preclinical studies support the antioxidant and cytoprotective effects of L-carnitine and its derivatives in experimental models with induced AP, showing improvements in oxidative stress markers, tissue integrity, and even pain-related outcomes. However, despite its promising biological profile, no human studies have yet assessed its role in AP. Further clinical research is essential to evaluate its therapeutic efficacy, optimal dosing, and safety profile.

2. Glutamine

Glutamine (Gln) is an abundant non-essential amino acid with antioxidant and immune functions. It is a crucial component in the synthesis of glutathione, as it acts as an indirect glutathione precursor, through glutamate [57]. In cases of acute illness, impaired Gln synthesis and an increased requirement for endogenous Gln are insufficient, thus justifying the reason for supplementation in severe AP. Moreover, Gln was specified as an obligatory parenteral nutrition (PN) supplement in critical care therapy starting at a dose of up to 10 g/day to a maximum dose of 30 g/day in critically ill patients, with maintenance throughout the hypercatabolic phase defined by increased CRP, decreased prealbumin levels, and negative nitrogen balance [58].

Dipeptidic Gln as alanine-glutamine dipeptide (N(2)-l-alanyl-l-glutamine) is a stable and highly soluble synthetic Gln product, superior to the unstable monopeptidic form, and should be administered intravenously at a dose of 0.3–0.6 g of Ala-Gln/kg/day, according to current guidelines [59].

The 2024 European Society for Parenteral and Enteral Nutrition (ESPEN) guideline on clinical nutrition in AP recommends that parenteral Gln should be supplemented at 0.20 g/kg/day in the form of L-glutamine when enteral nutrition (EN) is not feasible or contraindicated, mentioning a potentially increased bias risk in the included studies due to the small sample size, the heterogeneity of AP severity, and other confounding factors related to alternative therapeutic interventions [60].

In clinical studies on AP, Gln was used as a supplement to total parenteral nutrition (TPN) and demonstrated improved clinical outcomes: a decrease in the duration of TPN and hospitalization, reduced infectious complications, an increase in serum albumin and a decrease in serum CRP [61,62,63]. In addition to antioxidant properties, Gln has an important function in gut physiology by promoting enterocyte proliferation and contributing to the integrity of tight junctions. Also, it protects against apoptosis caused by stress and inflammation during pathologic conditions and thus contributes indirectly to decreased intestinal permeability and bacterial translocations, with definitive effects on SIRS [64].

A 2013 meta-analysis of four randomized controlled trials (RCTs) involving a total of 190 participants investigated the role of Gln dipeptide supplementation to standard PN or EN in severe AP. Results showed that the addition of Gln dipeptide was superior to standard PN or EN alone, without serious adverse effects. Outcomes included a reduced mortality rate, a reduced length of hospital stay, and a decreased rate of complications [65] (Table 2).

Another meta-analysis of RCTs on a total of 505 patients with AP concluded that Gln supplementation resulted in a significantly reduced risk of mortality (*p* < 0.001) and infectious complications (*p* = 0.009) but did not impact the length of hospital stay (*p* = 0.17). Notably, patients who received Gln in combination with other immunonutrients had significantly better outcomes [66].

A recent RCT showed that enteral Gln supplementation was able to improve gut function, nutritional status, and oxidative stress. A statistically significant reduction in IL-6 concentration was observed in the treatment group after completion (*p*  =  0.02). However, the rate of infected necrosis and in-hospital mortality did not improve with treatment [67].

A total of 433 participants from seven clinical RCTs were enrolled in meta-analyses: 218 patients in the early enteral nutrition (EEN) group and 215 patients in the Gln-supported EEN group (G+EEN). In contrast to EEN, G+EEN resulted in higher serum albumin (*p* < 0.01), lower serum high-sensitivity CRP (hsCRP) (*p* < 0.01), lower risks of mortality (*p* = 0.03), multiple organ dysfunction syndrome (*p* < 0.01), and shorter hospital stay (*p* < 0.01). No significant decrease in APACHE II scores, incidence of infection-related complications, and need for surgical procedures were observed. The meta-analysis concluded that G+EEN is a helpful adjuvant in the treatment of severe AP [68] (Table 2).

A 2022 meta-analysis including 30 RCTs and a total of 1 201 patients found that Gln supplementation at a dose of 0.4 g/kg/day enterally or parenterally in patients with AP was associated with decreased mortality and hospital stay and fewer complications (all *p* < 0.001), with superior outcomes in cases where the enteral route of administration was used regarding LoHS, except for mortality, where parenteral supplementation was superior in a subgroup analysis. As a secondary outcome, serum albumin and IgG levels significantly increased, while creatinine, CRP, IL-6, and IL-8 decreased [69] (Table 2). These findings suggest that Gln supplementation is extremely effective when added to TPN, as this strategy was more likely to reduce mortality and complication rates.

Another study on 78 patients aimed to investigate whether Gln and ulinastatin together, added to the conventional treatment (research group, RG), could reduce inflammation and enhance liver function in patients suffering from severe AP. The control group (CG) received ulinastatin only, along with conventional treatment. Following therapy, both groups showed an improvement in their clinical complaints. Compared to the CG, patients in the RG showed a quicker improvement in bowel sound recovery, first defecation, bloating, and stomach discomfort (*p* < 0.05). Following therapy, the RG had greater levels of IgM, IgA, and IgG than the CG (*p* < 0.05). Additionally, the RG showed significantly lower levels of IL6, TNF-α, and hsCRP than the CG (all *p* < 0.05). Following therapy, both groups’ liver function and amylase levels dropped, with the RG’s indexes being lower than the CG’s (*p* < 0.05) [70] (Table 2).

Glutamine supplementation, particularly in parenteral dipeptide form, has shown promising effects in patients with severe AP, including reduced mortality, fewer infectious complications, and improved nutritional and inflammatory markers. While some studies found no significant changes in hospital stay or APACHE II scores, data from multiple meta-analyses suggest a beneficial role, especially when combined with other immunonutrients or delivered early in enteral nutrition. Given its safety profile and physiological relevance in critical illness, glutamine remains a valuable adjunct in the nutritional management of severe AP. However, further research should define optimal formulations, dosing strategies, and patient selection.

3. Glutathione precursors

Glutathione depletion occurs invariably during the progression to severe AP and could be related to increased oxidative stress and impaired biosynthesis. Thus, glutathione precursors may improve antioxidant status in AP by restoring the glutathione pool.

Thus, glutathione precursors can improve the antioxidant status in AP through restoring glutathione reserves, which is essential for neutralizing oxidative stress and for preventing ferroptosis. Glutathione is a cofactor for glutathione peroxidase 4 (GPX4), the principal enzyme responsible for detoxifying lipid peroxides and inhibiting ferroptotic cell death, which is a key mechanism implicated in AP progression [71].

**N-acetylcysteine (N-ACC),** the N-acetyl derivative of the conditionally essential amino acid L-cysteine, can act as a reduced glutathione (GSH) precursor, which in turn is a well-known direct antioxidant. In conditions of oxidative stress and low nutritional intake, the depletion of endogenous cysteine and GSH occurs, and N-ACC can provide antioxidant activity, especially in the setting of liver injury (both acetaminophen and non-acetaminophen-induced), but also in AP [72]. Additionally, N-ACC exerts disulfide-breaking activity, explaining its mucolytic properties.

Several studies investigating the role of N-ACC supplementation in experimentally induced AP in rats showed beneficial results regarding the reduction in pancreatic tissue necrosis and the level of inflammation [73,74,75], improving pancreatic microvascular perfusion and reducing pancreatic damage and mortality.

Three clinical trials investigating the role of N-ACC supplementation for the prevention of post-ERCP AP (both prophylactic and post-procedural, in different doses, either oral or intravenous) failed to prove any beneficial effects on the incidence and severity of AP [76,77,78] (Table 2).

Only one RCT demonstrated a significantly reduced incidence of post-ERCP AP after N-ACC supplementation, but no impact on the length of hospital stay was identified [79] (Table 2). Another study demonstrated significantly reduced serum amylase after 24 h of N-ACC supplementation, but no significant impact on AP severity or length of hospital stay was observed [80] (Table 2).

A systematic review and dose–response meta-analysis of 26 controlled clinical trials, published in April 2023, included studies investigating the effect of N-ACC on antioxidant biomarkers. Total antioxidant capacity (*p* < 0.001), glutathione (*p* = 0.004), and catalase (*p* = 0.042) levels were all markedly elevated by N-ACC supplementation. Superoxide dismutase (*p* = 0.41), glutathione reductase (*p* = 0.21), and glutathione peroxidase (*p* = 0.57) levels, however, did not show any discernible improvement. Additionally, N-ACC supplementation had a stronger effect on raising GSH levels in participants with mean ages of up to 30 years, according to a dose–response analysis. The conclusion was that N-ACC may be regarded as a potential agent for increasing antioxidant capacity [81].

N-acetylcysteine has demonstrated promising antioxidant and cytoprotective effects in preclinical models of AP, with evidence of reduced pancreatic necrosis and improved microvascular perfusion. However, trials, including those targeting post-ERCP pancreatitis, have not shown significant improvements in clinical outcomes, with only isolated benefits reported in selected parameters such as enzyme levels or antioxidant capacity. Given all this, future clinical studies are needed and should focus on optimizing dosage, the route and timing of administration, and the identification of subgroups of patients who may benefit most, in order to better understand how effective N-ACC really is in treating acute pancreatitis.

**S-adenosyl methionine (SAMe)** is a highly bioactive metabolite of methionine, which increases glutathione S-transferase activity and works as a key defense mechanism against oxygen-reactive species. High plasmatic concentrations of S-adenosylmethionine in patients with AP suggest that the trans-sulfuration pathway towards glutathione synthesis may be disrupted, thus justifying the role of glutathione precursors in reducing oxidative stress in severe AP [82].

One clinical trial investigating the role of the intravenous administration of SAMe and N-ACC, within or after 15 h from the onset of symptoms, showed no impact on clinical outcomes, hospitalization stay, or mortality [83] (Table 3. However, the short supplementation period of only 24 h may have been insufficient to achieve a significant impact on clinical outcomes during this particular study. Further studies should be performed to better define its potential role and its therapeutic relevance in AP.

4. Melatonin

Melatonin is an amine derived from L-tryptophan and is metabolized to kynuramines, with one of the best-known being N-acetyl-N-formyl-5-methoxykynuramine. Formerly discovered as a pineal gland product, it is now known that it possesses antioxidant effects, with receptors being identified in the gastrointestinal tract and pancreas. While melatonin can stimulate pancreatic enzyme secretion, L-tryptophan and kynuramines have less effect on pancreatic exocrine function [84].

Multiple animal studies showed that melatonin, its precursor, and principal metabolite significantly attenuated AP through antioxidative and anti-inflammatory functions.

Also, experimental studies demonstrated that the administration of melatonin prior to the induction of AP significantly attenuated the intensity of the inflammation and was able to reduce pancreatic tissue damage [85,86,87,88]. Moreover, pinealectomy was shown to aggravate AP in rats, and intraperitoneal melatonin infusion significantly reduced pancreatic inflammation in the pinealectomized animals [89,90]. In pigs with experimentally induced AP, melatonin treatment significantly reduced acinar and fat tissue necrosis and pancreatic edema [91].

After experimentally induced pancreatic ischemia in rats, no histological sign of pancreatitis was observed 48 h after reperfusion in 80% of the animals treated with melatonin, supporting the fact that melatonin administration prevents pancreatic oxidative stress and protects against pancreatic tissue injury [92]. Another study demonstrated that melatonin has a positive effect on gut barrier dysfunction and bacterial translocation and reduces infection and early mortality rates in induced severe AP in rats [93].

Thus, melatonin might be a useful therapeutic option in severe acute pancreatitis, although the exact anti-inflammatory and antioxidant mechanisms are still not elucidated. Multiple studies demonstrated that melatonin was capable of limiting pancreatic damage in AP by protecting the structure of acinar cells, the modulation of apoptosis, and the promotion of tissue antioxidant enzyme activities [94,95]. Recent investigations have elucidated the involvement of melatonin in attenuating ferroptosis via its modulation of intercellular iron homeostasis, particularly through iron chelation [96].

A human study demonstrated that the serum melatonin concentration is closely related to the severity of AP and the BISAP score, with a value ≤ 28.74 ng/L being associated with an increased risk of developing severe AP. No correlation was found between the APACHE II score and serum melatonin [97]. Another study evaluating the dynamics of serum melatonin levels in the early phase of human AP found that concentrations in the first 24 h after onset were significantly higher in younger (<35 years old) than older (>35 years old) patients, with high levels playing a protective role by favoring a mild course of the disease. Interestingly, serum melatonin was significantly higher after AP onset in patients with mild compared to severe AP. There was no correlation between melatonin levels and the etiology of pancreatitis [98]. To the best of our knowledge, a single double-blind randomized study on the role of melatonin supplementation in human AP has been carried out so far and showed that the addition of melatonin to indomethacin 1 h before ERCP decreased the rate of post-ERCP AP compared to indomethacin alone [99] (Table 2).

While the beneficial effects of exogenous melatonin have already been demonstrated in multiple experimental animal studies and one human study so far, endogenous melatonin could be a native mechanism protecting the pancreas from oxidative stress and acute damage, given that pinealectomy resulted in the exacerbation of AP and that low melatonin plasma levels were associated with an increased risk of severe AP [100].

5. Agomelatine

In a recent study, the melatonin receptor agonist agomelatine, an antidepressant, protects against the model of AP induced by cadmium in rats by attenuating inflammation and oxidative stress and modulating the Nrf2/HO-1 pathway. This study might indicate that melatonin has a particular mechanism of action in AP besides its non-specific antioxidant properties [101].

Melatonin showed significant positive results in experimental settings, including a pig model, and it may act as a supportive therapy in patients at high risk for AP to prevent pancreatic inflammation. However, the exogenous supplementation with melatonin has not yet been properly studied in human AP.

In conclusion, melatonin has demonstrated consistent protective effects in experimental models of AP, including a reduction in tissue damage, the modulation of inflammatory responses, and an improvement in gut barrier integrity. These findings suggest a potential therapeutic role, particularly in the early stages of pancreatic injury. While observational studies have linked lower serum melatonin levels to greater disease severity, the clinical relevance of supplementation remains to be studied. Further research is necessary to clarify its mechanisms of action and to evaluate its efficacy and safety in human AP.

Table 2 summarizes the main characteristics of studies investigating the effect of amino acid and derivative supplementation in AP.

**Table 2:** Amino acid and derivative supplementation in acute pancreatitis.

Antioxidant	First Author, Year	Study Design and Aim	Dose and Duration of Supplementation	No. of Participants	Outcomes	Adverse Effects
L-carnitine		No human studies.			Significant impact on reducing oxidative stress and increasing antioxidant function in experimentally induced AP.	
Glutamine	Zhong et al., 2013 [65]	*Meta-analysis of 4 RCTs.* To evaluate the efficacy of IV Gln in severe AP.	Gln dipeptide ranging from 0.4 g/kg to 20 g/day IV for ≥ 7 days	190	Decreased mortality (*p* = 0.01), LoHS (*p* < 0.001), and complication rate (*p* = 0.006).	No significant adverse effects
	Jiang et al., 2020 [68]	*Meta-analysis of 7 RCTs.* To assess if Gln-supported EEN has therapeutic benefits in severe AP.	0.1 to 0.5 g/kg/day for 5 to 14 days	433 (EEN group: 218 patients; Gln + EEN group: 215 patients)	Higher serum albumin (*p* < 0.01), lower serum hsCRP (*p* < 0.01), lower risk of mortality, and shorter LoHS (*p* < 0.01). No significant impact on APACHE II scores, incidence of infection-related complications, or surgical interventions.	None reported ^1^
	Dong et al., 2022 [69]	*Meta-analysis of 30 RCTs.* To evaluate the efficacy of Gln supplementation in severe AP.	0.4 g/kg parenterally or enterally	1 201	Pooled data showed decreased mortality in PN group (*p* = 0.001), but not in EN group (*p* = 0.25); LoHS and complications were significantly reduced (both *p* < 0.001); significantly increased serum albumin and IgG (both *p* < 0.00001) and decreased serum creatinine (*p* = 0.008), CRP (*p* < 0.0001), IL-6, and Il-8 (both *p* < 0.00001), and decreased bloating recovery time (*p* < 0.00001).	None reported
Glutamine + Ulinastatin	Zhao et al., 2022 [70]	To investigate if Gln and ulinastatin supplementation can reduce inflammation and enhance liver function in patients suffering from severe AP.	In total, 500 mL of 5% glucose injection with ulinastatin (twice daily; 100 mL: 5 g). Gln (100 mL: 20 g) once a day via intravenous drip at a dose of 20 g each.	78	Quicker recovery of bowel sounds, first defecation, bloating, and abdominal pain; higher levels of IgM, IgA, and IgG (all *p* < 0.05). Significantly lower levels of IL-6, TNF-α, and hsCRP (all *p* < 0.05).	None reported
N-ACC	Katsinelos et al., 2005 [76]	*Double-blind placebo-controlled.* To assess the efficacy of N-ACC in the prevention of post-ERCP AP.	IV loading dose: 70 mg/kg 2 h before ERCP and 35 mg/kg at 4 h intervals for 24 h after ERCP.	249	No beneficial effects on AP incidence and severity.	None reported
	Milewski et al., 2006 [77]	*RCT.* To assess the efficacy of N-ACC in the prevention of post-ERCP AP.	In total, 600 mg orally at 24 and 12 h before ERCP and 600 mg of IV b.i.d for 48 h after ERCP.	55	No beneficial effects on AP incidence. No impact on baseline and post-ERCP serum and urine amylase.	None reported
	Alavi Nejad et al., 2013 [79]	*Double-blind, placebo-controlled RCT.* To assess the impact of N-ACC on post-ERCP AP incidence and LoHS.	1200 mg orally 2 h before ERCP.	100	Significantly reduced incidence of AP (10% vs. 28% for placebo, *p* = 0.02); no significant impact on LoHS (*p* = 0.8).	None reported
	Pavel et al., 2019 [78]	*Single-blinded RCT.* To assess the efficacy of indomethacin in various doses with or without N-ACC for the prevention of post-ERCP AP.	Group 1: 600 mg of N-ACC IV 15 min before ERCP + indomethacin 50 mg IR before and after ERCP. Group 2: indomethacin alone 50 mg IR before and after ERCP. Group 3 (standard regimen): indomethacin 100 mg IR after ERCP.	186	No beneficial effects of N-ACC supplementation; no significant difference in AP incidence btw. the 3 groups (*p* = 0.24); similar efficacy of split dose vs. standard regimen.	No adverse effects
	Sivakumar et al., 2024 [80]	*Prospective observational study.* To evaluate the impact of N-ACC supplementation on AP resolution and LoHS.	IV infusion 1 g/5 mL + 25 mL of normal saline, followed by oral tablet 600 mg b.i.d.; evaluation at 24 and 48 h.	65	Significantly reduced serum amylase at 24 h (*p* = 0.01); no impact on serum lipase (*p* = 0.1). No significant difference in Ranson score (*p* = 0.4); significant impact on APACHE II score at 24 h (*p* = 0.04) and 48 h (*p* = 0.01). No impact on LoHS (*p* = 0.4).	None reported
SAMe + N-ACC	Sharer et al., 1995 [83]	*RCT.* To assess the effect of antioxidants on clinical outcomes in mild and severe AP.	SAMe 43 mg/kg + N-ACC 300 mg/kg within 24 h from admission (within or after 15 h from symptom onset).	79	No impact on complication rate, LoHS, or mortality.	None reported
Melatonin	Sadeghi et al., 2019 [99]	*Double-blind placebo-controlled RCT.* To determine whether melatonin reduces the rate of post-ERCP AP.	In total, 3 mg orally + indomethacin 100 mg IR 1 h before ERCP.	411	Significantly decreased post-ERCP AP rate (9.3% vs. 15% for placebo, *p* = 0.042); lower levels of lipase/amylase (*p* = 0.032 and *p* = 0.041, respectively).	No adverse effects

^1^ no data about adverse effects was mentioned in the study; RCT = randomized controlled trial; CCT = controlled clinical trial; AP = acute pancreatitis; LoHS = length of hospital stay; CRP = C-reactive protein; N-ACC = N-acetylcysteine; SAMe = S-adenosyl methionine; EEN = early enteral nutrition; hsCRP = high-sensitivity C-reactive protein; SOD = superoxide dismutase; GPx = glutathione peroxidase; b.i.d. = twice a day; ERCP = endoscopic retrograde cholangiopancreatography; IR = intrarectal.

#### 3.1.3. Phytonutrients and Selenium

In addition to antioxidants, many phytonutrients were also studied in AP, and their anti-inflammatory and antioxidant effects may be beneficial due to their multiple protective abilities and the probability to attenuate pancreatic cell injury by reducing inflammatory response, as was reported in a recently published comprehensive review [102]. Another study found that phytonutrients such as flavonoids, lignans, anthraquinones, and alkaloids have a role in maintaining the balance between necrosis and apoptosis in AP [103].

1. Organosulfur compounds

Organosulfur compounds represent a class of naturally occurring phytochemicals found in garlic, onions, and cruciferous vegetables, which exhibit antioxidant and anti-inflammatory effects demonstrated in experimental AP.

Dimethyl trisulfide (DMTS) may have high cytoprotective properties, being capable of the modulation of oxidative stress by improving the activity of endogenous antioxidant enzymes and diminishing ROS accumulation. In a cerulein-induced model of AP in rats, DMTS significantly reduced serum amylase and lipase levels, diminished pancreatic myeloperoxidase activity, and improved histopathologic injury by reducing neutrophil infiltration and lipid peroxidation. These outcomes may suggest that DMTS can have protective effects through antioxidant and anti-inflammatory mechanisms. Further studies are needed to confirm its therapeutic value in human AP [104].

Diallyl disulfide (DADS), an organosulfur compound, has shown positive effects in inflammatory and oxidative conditions, including experimental AP. In cerulein-induced AP models, DADS reduced pancreatic edema, neutrophil infiltration, and pro-inflammatory cytokine levels (TNF-α and IL-6). Also, DADS reduced oxidative stress by increasing SOD activity and reducing MDA levels in pancreatic tissue. These outcomes highlight its role in reducing inflammatory signaling and restoring redox balance in acinar cells. DADS may be an adjuvant in oxidative stress-related AP therapy [105].

Sulforaphane is another organosulfur phytonutrient with important antioxidant and cytoprotective effects. In murine models of AP, sulforaphane administration significantly reduced serum pancreatic enzyme levels and ameliorated histological pancreatic damage. Sulforaphane activates the nuclear factor erythroid 2-related factor 2 (Nrf2) signaling pathway and up-regulates phase II detoxification enzymes (heme oxygenase-1 (HO-1) and glutathione S-transferase), thereby enhancing cellular resistance against oxidative injury. Sulforaphane also modulates autophagy and reduces inflammatory mediators, proving to be a promising dietary compound with therapeutic benefits in oxidative stress-mediated pancreatic diseases [106].

2. Resveratrol

Resveratrol is a polyphenolic antioxidant found in high concentrations in grapes, blueberries, plums, and apples. After ingestion in the form of *trans*-resveratrol, it is rapidly metabolized by colonic flora and converted to a more biologically active form, dihydro-resveratrol, which exerts many properties: anti-inflammatory, antioxidant, and inhibition of platelet aggregation [107].

Resveratrol’s immunomodulatory and antioxidant qualities might offer a viable chemo-preventive strategy for AP prevention. Resveratrol pretreatment has been shown to decrease hyperamylasemia, hyperlipidemia, and histological damage brought on by cerulein therapy [108].

In a recent study, a novel synthetic analog of natural resveratrol [(R)-4,6-dimethoxy-3-(4-methoxy phenyl)-2,3-dihydro-1H-indanone] showed positive results on experimental AP by significantly decreasing the inflammatory response [109]. In rat cerulein-induced AP, resveratrol showed chemo-preventive and immunomodulatory effects and was able to partially reverse the abnormal calcium signal in acinar cells [108]. In the same animal model, the oral administration of resveratrol significantly inhibited glutathione depletion [110] and was able to restore plasma glutathione peroxidase concentrations [111].

An effect of resveratrol is to restore the total antioxidant status to a normal level in patients with AP by suppressing TNF-induced lipid peroxidation and by immunomodulatory and cytoprotective effects [112]. In vitro, the resveratrol treatment of isolated human pancreatic islets significantly reduced oxidative stress and improved their survival and viability [113].

Some animal studies indicate the physiological roles of resveratrol: it reduces the levels of MDA, increases the levels of SOD, endothelial nitric oxide synthase, and inducible nitric oxide synthase, and decreases the level of the nicotinamide adenine dinucleotide phosphate (NADPH) oxidase system. Resveratrol can diminish pancreatic damage by regulating the generation of inflammatory molecules, improving the anti-oxidative defense system, and modulating the level of calcium in pancreatic cells in rats [107].

Resveratrol has been shown in several investigations to shield pancreatic acinar cells against leukocyte infiltration and autodigestion. By lowering lipid peroxidation and enhancing the antioxidant defense system through Nrf2 pathway activation, it also preserves the structural integrity of cell membranes [114].

According to a recent animal study, resveratrol pretreatment can restrict the inflammatory response by blocking NF-κB signaling, minimizing oxidative stress, and ameliorating pancreatic tissue damage [115].

In an animal study that evaluated the effects of beta-carotene, resveratrol, and L-arginine on induced AP in rats, the outcomes showed that resveratrol and beta-carotene treatments significantly raised glutathione levels and decreased malondialdehyde levels (*p*  < 0.01 for both). Preserved β+ Langerhans islet size (*p*  < 0.01), the decreased expression of NF-κB, TNF-α, and IL-1β, decreased levels of Bax and Caspase-3, and elevated levels of Bcl-2 were also seen in the groups that had received resveratrol and beta-carotene [116].

Taking into account the fact that the active form of resveratrol is a microbial metabolite, patients unresponsive to trans-resveratrol may lack proper microbial strains and could thus benefit from the oral intake of the hydrogenated product, presuming that this strategy revealed positive results in animal studies [110,117].

After reviewing a total of 50 studies on animal models (33 for acute pancreatitis, 1 for chronic pancreatitis, and 16 for pancreatic cancer), a meta-analysis published in 2025 found that, in acute pancreatitis models, resveratrol showed notable improvements in lung injury (lung histopathology and myeloperoxidase), oxidative biomarkers (malondialdehyde and superoxide dismutase), inflammatory markers (TNF-α, IL-1β, IL-6, and pancreatic myeloperoxidase), and pancreatic histopathology scores and pancreatic function parameters (serum amylase and lipase). Resveratrol significantly decreased the weight and volume of tumors in models of pancreatic cancer. Notably, 20–105 mg/kg with 3–9 doses was the ideal single dosage [118].

Resveratrol shows strong antioxidant and anti-inflammatory effects in experimental models of AP, improving both pancreatic and systemic markers of injury. Despite promising preclinical data, no clinical studies are currently available. Future research should focus on confirming its efficacy in humans and clarifying optimal dosing, formulation, and the role of gut microbiota in its activation.

3. Lycopene

Lycopene, along with lutein and zeaxanthin, is a major dietary non-provitamin A carotenoid that is found in high concentrations in tomato-based products, being unaffected by food processing. As it cannot be synthesized in the human body, it must be supplemented daily [119].

A recent study showed that lycopene is the leading carotenoid in plasma and tissues, being the most potent antioxidant, second only to astaxanthin [120]. It is an important deactivator of ROS and can enhance antioxidant enzyme activities. Lycopene can act on free radicals (including mitochondrial ROS) in the same way as hydrogen peroxide [121]. Furthermore, it can regulate glutathione levels in hepatocytes, significantly preventing mitochondrial DNA damage in in vitro studies [122], and exerts beneficial effects regarding the prevention of multiple conditions, including cancer, as demonstrated by a recent systematic review and meta-analysis [123].

By boosting the cellular antioxidant defense system, lycopene has been shown to improve the status of enzymatic (catalase, superoxide dismutase, and peroxidase) and nonenzymatic antioxidants (vitamins C and E) [124]. In systems that generate singlet oxygen, low doses of lycopene function as an antioxidant; yet, in systems that generate peroxide, it works as a pro-oxidant [125].

Regarding AP, multiple experimental animal studies demonstrated the beneficial effects of lycopene supplementation, with potent antioxidant and anti-inflammatory effects occurring mostly in a dose-dependent manner. In cerulein-induced AP in rats, lycopene decreased intracellular ROS and IL-6 expression in pancreatic acinar cells [126,127].

At a dose of 50 mg/kg, lycopene inhibited neutrophil infiltration and lipid peroxidation [128]. At the same dose, administered once daily for 10 days before injection of L-arginine, it significantly prevented AP through anti-inflammatory and antioxidant effects: the down-regulation of inducible NO synthase gene expression, a significant reduction in TNF-α and MPO activity, and an increase in the pancreatic GSH level [129].

A recent study found that circulating levels of antioxidants, including carotenoids, tend to decrease over the course of the transition from early CP to definite CP; plasma lycopene, in particular, was 21.5 and 14.5 μg/dL, respectively, compared to 36.6 μg/dL for controls [130], which is possibly related to malabsorption, insufficient intake, or high demand due to increased oxidative stress.

Lycopene has shown strong antioxidant and anti-inflammatory properties in experimental models of AP, primarily in a dose-dependent manner. While preclinical data are promising, no clinical studies have yet evaluated its efficacy. Further research should be conducted to determine its potential therapeutic role in AP and to assess appropriate dosing and safety in human subjects.

4. Quercetin

Quercetin is a natural antioxidant from the flavonoid group of polyphenols, present in high concentrations in onions, apples, and other plant-based foods. Multiple studies mentioned several beneficial biological activities of quercetin: the scavenging of ROS, an increasing glutathione level, anti-inflammatory, anti-tumor, and immunostimulant properties [131,132]. Recently, quercetin gained popularity after many clinical trials, including RCTs, and demonstrated the therapeutic and prophylactic effects of its supplementation in patients with a SARS-CoV2 infection at a dose of up to 1000 mg/day [133,134,135].

Multiple studies on cerulein-induced AP in mice demonstrated that quercetin supplementation was able to attenuate the severity of AP by reducing pancreatic neutrophil infiltration, IL-1β, IL-6, and TNF-α production, and an increasing reduced vs. oxidized glutathione ratio [136,137,138,139]. Also, quercetin supplementation demonstrated significant antioxidant properties in L-arginine-induced AP in rats in a dose-dependent manner [140].

A study investigated the impact of quercetin glycosylation on improving its pharmacologic activities and found that the glycosylated derivative quercetin 3-*O*-xyloside had significant and superior antioxidant and anti-inflammatory activity in cerulein-induced AP in mice by suppressing intracellular ROS production and the endoplasmic reticulum stress response, compared to the non-glycosylated quercetin aglycone [141].

Quercetin’s anti-inflammatory effect was further demonstrated. By lowering the levels of inflammatory mediators, such as TNF-α, and reducing the activation of the p38 MAPK signaling pathway, quercetin attenuated cerulein-induced AP [139].

Quercetin supplementation has shown significant antioxidant and anti-inflammatory effects in experimental models of AP, primarily by modulating cytokine production, oxidative stress, and stress signaling pathways, but its effect on human AP has yet to be addressed. Modified forms, such as glycosylated quercetin, appear to enhance its efficacy. Quercetin supplementation showed beneficial effects in experimentally induced AP. Although quercetin was termed safe for use by the USFDA, it may alter the bioavailability of some antibiotics, antivirals, and steroid anti-inflammatory drugs, and these interactions should be better defined before adding quercetin to a therapeutic regimen [142].

5. Curcumin

Curcumin is a polyphenol belonging to the group of curcuminoids and the principal phytochemical found in turmeric, a member of the ginger family. In addition to being used as a food flavoring and food coloring agent (due to its bright yellow color), curcumin is a potent anti-inflammatory and antioxidant molecule, with demonstrated activity at the cellular level by targeting multiple signaling pathways [143,144].

Curcumin can neutralize free radicals by modulating the activity of GSH, catalase, and SOD enzymes and inhibit ROS-generating enzymes such as lipoxygenase and cyclooxygenase, thus combating inflammation and oxidative stress [145].

A systematic review and meta-analysis of RCTs demonstrated that curcumin was able to block NF-κB, a nuclear transcription factor that regulates the effect of TNF-α, thus lowering its concentrations, with potential efficacy against several inflammatory diseases, including inflammatory bowel disease [146]. Due to its low bioavailability, curcumin should be supplemented along with piperine, an effective bioenhancer that increases its absorption and inhibits its hepatic and intestinal glucuronidation. Clinical studies showed that there are no significant side effects in humans up to doses of 12 g/day, except diarrhea, mild nausea, and headache. However, the Allowable Daily Intake value of curcumin is 0–3 mg/kg body weight [147].

The benefits of curcumin supplementation in experimentally induced AP have been demonstrated in several animal models. Curcumin was able to significantly reduce the inflammatory response, lowering serum IL-6, CRP, and TNF-α, reducing pancreatic injury, and lowering the levels of pancreatic enzymes [148,149,150]. Moreover, it was shown to ameliorate the deleterious effects of AP on other organs, as indicated by a decrease in aminotransferase levels and a reduction in ascites volume [151], probably by decreasing the activity of enzymes responsible for oxidative stress [152]. A single-blind, randomized, placebo-controlled pilot study evaluating the effects of 6-week curcumin supplementation on the clinical evolution and oxidative stress biomarkers in 20 patients with chronic tropical pancreatitis demonstrated significantly reduced erythrocyte MDA levels as a marker of lipid peroxidation and increased GSH levels compared to placebo. However, it showed no impact on pain [153] (Table 3).

A randomized controlled trial from 2023 sought to investigate whether nano-curcumin, as an anti-inflammatory medication, would be useful in patients with mild and moderate AP. Researchers investigated the effectiveness of giving nano-curcumin twice a day for two weeks to patients with mild-to-moderate AP. They discovered that it was a safe adjuvant therapy that shortened the length of hospital stay and reduced the need for analgesics [154] (Table 3). Other clinical studies on the efficacy of curcumin in AP evolution are lacking.

6. Selenium

Selenium is a trace mineral found in meat, fish, shellfish, eggs, onions, and cruciferous vegetables, serving as a crucial component of selenoproteins with antioxidant and immunomodulatory functions, such as glutathione peroxidase [155]. It also plays a role in thyroid hormone metabolism and male fertility and has been shown to enhance DNA repair mechanisms and endothelial antioxidant defense, offering protection against cardiovascular disease [156].

In both chronic pancreatitis and acute pancreatitis (AP), serum selenium levels are significantly lower than in healthy individuals and are inversely related to disease severity and glutathione peroxidase (GPx) activity, indicating compromised antioxidant capacity [157,158].

Although a few case reports have demonstrated transient improvements in antioxidant enzyme levels and inflammatory markers following selenium supplementation in severe AP [159], clinical data remain limited and inconsistent (Table 3).

Several randomized controlled trials in critically ill patients with SIRS and sepsis—conditions that share pathophysiological features with severe AP—have evaluated high-dose selenium supplementation. While plasma selenium and GPx levels increased post-treatment, there was no significant impact on mortality or major inflammatory markers compared to the control groups [160,161,162,163] (Table 3). A meta-analysis of 21 RCTs similarly found no benefit in terms of mortality, renal outcomes, or ICU stay duration [164]. In the context of AP, a randomized, blinded trial showed no benefit of selenium supplementation on clinical outcomes after a 90-day follow-up [165], and additional studies reported only modest biochemical improvements, such as increased GPx and decreased MDA activity, without consistent changes in SOD levels [166] (Table 3). The prophylactic administration of selenium in ERCP-induced AP also failed to demonstrate clinical benefits [167] (Table 3).

Experimental studies continue to highlight selenium’s mechanistic relevance in AP. In animal models, both conventional and nano-selenium significantly attenuated oxidative stress, inflammatory cytokine expression, and histopathological pancreatic damage [168].

In cerulein + LPS-induced severe AP, selenium was shown to activate Nrf2/HO-1 signaling while inhibiting the MAPK, NF-κB, and STAT3 pathways, thereby reducing pancreatic and pulmonary injury and decreasing the serum levels of amylase, lipase, and pro-inflammatory mediators [169].

Taken together, these findings support a potential antioxidant role for selenium in AP, particularly in reducing oxidative injury and modulating inflammatory signaling pathways. However, despite promising preclinical evidence, the majority of clinical trials have not demonstrated a significant impact on major clinical outcomes, thus limiting their current therapeutic utility in routine practice. Additional clinical data from well-designed, targeted trials are needed to validate selenium’s role in AP management [170,171].

Table 3 summarizes the main characteristics of studies investigating the effects of phytonutrients and selenium supplementation in AP.

**Table 3:** Phytonutrients and selenium supplementation in acute pancreatitis.

Antioxidant	First Author, Year	Study Design and Aim	Dose and Duration of Supplementation	No. of Participants	Outcomes	Adverse Effects
Resveratrol, Lycopene, Quercetin		No human studies.			Significant anti-inflammatory and antioxidant effects in experimentally induced AP.	
Curcumin	Durgaprasad et al., 2005 [153]	*Single-blind placebo-controlled RCT.* To evaluate the effect of curcumin on pain and markers of oxidative stress in tropical pancreatitis.	In total, 500 mg of curcumin + 5 mg of piperine orally for 6 weeks.	20	Significant reduction in erythrocyte MDA and significant increase in GSH levels; no improvement in pain.	None reported ^1^
	Chegini et al., 2023 [154]	*Double-blind, parallel-arm RCT.* To determine if individuals with mild-to-moderate AP respond well to nano-curcumin as an anti-inflammatory medication. LoHS was the primary endpoint.	Two doses of nano-curcumin (40 mg) or placebo, daily for two weeks.	42	It improved the overall appetite score (*p* = 0.049), significantly decreased LoHS (*p* = 0.006), and decreased the requirement for analgesics over time (*p* = 0.001).	No adverse effects
Selenium (Se)	Wollschläger et al., 1997 [166]	*Single-arm clinical study.* To investigate the effect of high-dose Se on oxidative status in AP.	Day 1: 2000 µg (1000 µg IV bolus + 1000 µg prolonged infusion over 10 h). Days 2–5: 1000 µg/day continuous infusion. Days 6–8: 1000 µg orally t.i.d.	16	Significant increase in serum Se; moderate increase in Gpx activity, significant decrease in MDA activity; SOD remained unchanged.	None reported
	Wollschläger et al., 1999 [167]	*Prospective RCT.* To investigate the effect of prophylactic Se on clinical and biological outcomes of post-ERCP AP.	Day before ERCP: 1000 µg IV bolus + 1000 µg prolonged infusion over 5 h. Day of ERCP: 1000 µg prolonged infusion over 5 h.	40	No impact on symptoms and need for analgesics; no significant impact on AP incidence or antioxidant status (GPx and MDA serum levels).	None reported
	Lindner et al., 2004 [165]	*Double-blind placebo-controlled RCT.* To investigate the effect of Se on clinical and paraclinical parameters of AP.	Day 1: 2000 µg IV. Days 2–5: 1000 µg IV. Day 6 until discharge: 300 µg IV.	67	No significant effect on clinical course of AP; median LoHS24 days for treatment group vs. 26 days for controls; no significant impact on oxidative status.	None reported
	Kočan et al., 2010 [159]	*Case report.* To investigate the impact of selenium supplementation inflammation and antioxidant markers in severe AP with septic shock.	Continuous infusion of 750 mg/24 h during the next six days after septic shock; 100 mL of alanyl-Gln/day was also added.	Case report	Glutathione peroxidase activity, an antioxidant enzyme, and other inflammatory indicators decreased.	None reported

^1^ no data about adverse effects were mentioned in the study; RCT = randomized controlled trial; MDA = malondialdehyde; GSH = glutathione; AP = acute pancreatitis; GPx = glutathione peroxidase; SOD = superoxide dismutase; ERCP = endoscopic retrograde cholangiopancreatography; LoHS = length of hospital stay.

#### 3.1.4. Omega-3 Polyunsaturated Fatty Acids (PUFAs)

Omega-3 PUFAs, represented by eicosapentaenoic acid (EPA), docosahexaenoic acid (DHA), and alpha-linolenic acid (ALA) are long-chain fatty acids exerting anti-inflammatory and antioxidant properties through multiple pathways: the suppression of lipid peroxidation and the enhancement of mitochondrial fatty acid oxidation by PPARγ activation, the inhibition of arachidonic acid (an omega-6 fatty acid)-derived pro-inflammatory eicosanoids like prostaglandin E2 and leukotriene B4 [172], and the regulation of platelet function by preventing platelet activation and aggregation [173,174].

Additionally, EPA and DHA are precursors to specialized mediators, such as resolvins and protectins, which actively resolve inflammation and promote tissue repair. Preclinical models demonstrate that omega-3 supplementation enhances resistance to oxidative damage and reduces pancreatic inflammation by modulation of the cytokine response—increasing anti-inflammatory cytokines like IL-10 and reducing IL-6 and IL-8, which correlates with histopathological and clinical improvement [175,176,177]. When compared to omega-6 and omega-9 PUFA-infused parenteral nutrition, only omega-3 infusion reduced the severity of histopathologic changes and decreased lipid peroxidation in induced hemorrhagic AP in rats [178].

Emerging clinical evidence highlights the therapeutic potential of omega-3 fatty acids in improving clinical outcomes in severe AP and critically ill patients. A systematic review and meta-analysis including 783 patients with SIRS, a hallmark of severe AP, concluded that omega-3 supplementation significantly reduced the mortality rate and hospital stay [179].

The impact of omega-3 supplementation on organ failure represents one of the most significant clinical findings. A meta-analysis of 85 patients with AP revealed a 67% reduction in new-onset organ failure with omega-3 therapy (OR 0.33, 95% CI 0.12–0.93, *p* = 0.04), with this effect being attributed to the attenuation of endothelial dysfunction and microcirculatory impairment, critical drivers of multi-organ failure in severe AP [180] (Table 4).

A meta-analysis of eight RCTs showed that both enteral and parenteral omega-3 supplementation resulted in a significantly reduced risk of mortality and infectious complications [181] (Table 4). Omega 3 parenteral supplementation, compared to soybean oil-based solution (high in omega-6), significantly increased plasma IL-10 at day 6 (*p* = 0.04); HLA-DR expression on monocytes, as a marker of immune competence, was significantly higher expressed in the omega-3 group compared to the omega-6 group (*p* = 0.01) [182]. Patients supplemented with omega-3 had a significantly higher serum EPA concentration (*p* < 0.01), lower CRP level (*p* < 0.05), and shorter interval of continuous renal replacement therapy (*p* < 0.05) [183].

An RCT of 45 patients with predicted severe AP demonstrated that omega-3 supplementation significantly reduced organ dysfunction scores—SIRS (*p* = 0.03), MODS (*p* = 0.03), SOFA (*p* = 0.004), the rate of new organ failure (*p* = 0.07), critical care admission (*p*= 0.06), critical care stay (*p* = 0.03), and total hospital stay (*p* = 0.04). In total, 50% of patients in the control group developed sepsis compared with 36% in the omega-3 group, but the difference was not statistically significant (*p* = 0.36). Inflammatory markers tended to be significantly reduced in the omega-3 group, and no adverse effects were noted [184].

In a study of 220 patients, those receiving omega-3 infusion (1.5 g/kg daily) exhibited a 36.5% reduction in CRP levels by day 7 compared to those receiving octreotide (*p* < 0.01). Moreover, the omega-3 cohort had a significantly lower incidence of pancreatic necrosis, infection rate, and shorter hospital stay, attributed to a faster resolution of abdominal pain and oral feeding tolerance. Organ failure and mortality rates were lower in the omega-3 group, but the results were not statistically significant [185] (Table 4).

Omega-3 also exhibits a beneficial impact on nutritional status. The ESPEN clinical guidelines for nutrition recommend the use of omega-3 PUFAs in patients with advanced cancer undergoing chemotherapy and at risk of weight loss or malnutrition [186], with proposed dosing recommendations of 2–5 g per day (in a 2:1 ratio of EPA to DHA) [187]. In a study on pancreatic cancer patients supplemented with oral PUFAs (2.2 g EPA/day), omega-3 stabilized body weight (1 kg increase over 3 weeks) and improved appetite scores and performance status [188], with further evidence supporting these findings [189]. Similarly to pancreatic cancer, AP induces a hypercatabolic state, exacerbating malnutrition and muscle wasting, and omega-3 supplementation may play a role in mitigating AP-related cachexia and improving nutritional status and recovery in severe AP.

Moreover, omega-3 PUFAs may enhance the efficacy of enteral nutrition by modulating gut-associated lymphoid tissue and reducing intestinal permeability [190]. Trials incorporating omega-3 into early enteral feeding protocols report fewer infectious complications and shorter ICU stays, as well as improved enteral nutrition tolerance and earlier weaning from PN in critically ill patients [191], underscoring its integrative potential in nutritional support.

Omega-6-enriched PN (mainly derived from conventional soybean oil) has a potential pro-inflammatory effect. In recent years, new mixed-oil formulas of modified soybean oil enriched in oleic acid (an omega-9 MUFA), medium-chain triglycerides, and olive oil have gained popularity as viable low-omega-6 alternatives for parenteral nutrition, with evidence showing improved clinical outcomes [192]. The recent global discourse on lipids at the PN Summit agreed that incorporating omega-3 into intravenous lipid emulsions yields significant clinical advantages without any indicators of adverse effects, based on robust biological justification and prevailing clinical data [193]. However, more informative trials are needed for strong consensus recommendations regarding optimal dosage and the duration of administration.

While parenteral supplementation with omega-3 showed superior results in some studies, there is also an added beneficial effect of omega-3 enteral supplementation on gut homeostasis and an improved tolerance of early enteral feeding, which in turn is known to be superior to PN with respect to complication rates and mortality [194]. The combined enteral and parenteral supplementation of omega-3 could have a synergistic effect on AP outcomes, but clinical trials investigating this issue have not yet been conducted, so arguments on this aspect are still premature.

Table 4 highlights the main results of clinical studies investigating the effect of omega-3 supplementation in AP.

**Table 4:** Omega-3 supplementation in acute pancreatitis.

First Author, Year	Study Design and Aim	Dose and Duration of Supplementation	No. of Participants	Outcomes	Adverse Effects
Lei et al., 2015 [181]	*Meta-analysis of 8 RCTs.* To investigate the impact of omega-3 supplementation on mortality, infectious complications, LoHS and ICU stay in severe AP.	Doses varied from 0.15 to 0.2 g/kg/day in 4 studies (PN) to 2.84 g/day in 1 study (EN). One study administered omega-3 enterally in combination with Gln and arginine. Duration: 3–15 days.	364	Pooled data from 6 RCTs (*n* = 264) showed that omega-3 significantly reduced mortality (RR 0.35; 95% CI 0.16 to 0.75, *p* < 0.05). In a subgroup analysis, the beneficial effect was statistically significant only in the PN group (RR 0.37; 95% CI 0.16 to 0.86; *p* < 0.05), while the EN group had no significant benefit (RR 0.28; 95% CI 0.05 to 1.61; *p* > 0.05). Pooled data from 5 RCTs (*n* = 219) revealed a significant reduction in infectious complications compared to controls (RR 0.54; 95% CI 0.34 to 0.85; *p* < 0.05), with similarly different results for PN (RR 0.50; 95% CI 0.28 to 0.9; *p* < 0.05) vs. EN (RR 0.62; 95% CI 0.3 to 1.28; *p* > 0.05). Pooled data from 5 RCTs (*n* = 203) showed a significant reduction in LoHS compared with controls (MD −6.50; 95% CI −9.54 to −3.46, *p* < 0.05), with similarly different results for PN (MD −8.13; 95% CI −10.39 to −5.87, *p* < 0.05) vs. EN (MD −0.82; 95% CI −12.44 to 10.79, *p* > 0.05) in the subgroup analysis. Pooled analysis of 3 RCTs (*n* = 116) showed no significant effect on ICU stay until exclusion of EN-supplemented patients (MD, −4.40; 95% CI −6.13 to −2.66, *p* < 0.05).	None reported. ^1^
Wolbrink et al., 2020 [180]	*Meta-analysis of 5 RCTs.* To evaluate the safety and efficacy of omega-3 supplementation in moderate and severe AP within 48 h of admission. Primary outcome: in-hospital mortality. Secondary outcomes: rate of new-onset organ failure and infectious complications.	EN (1 study): 3.3 g/day vs. SMC. PN (4 studies): 0.2 g/kg/day (up to 10 g/day) vs. SMC or soybean oil. Duration: 5–7 days.	229	Pooled data from 4 RCTs (*n* = 169) showed a non-significant reduction in mortality compared to controls (OR 0.37, 95% CI 0.09–1.56, *p* = 0.18). Based on 2 RCTs (*n* = 85), there was a lower risk for new-onset organ failure in the omega-3 group (OR 0.33, 95% CI 0.11–0.93, *p* = 0.04), and a trend towards a lower risk of infectious complications based on pooled data from 4 RCTs (*n* = 169) (OR 0.53, 95%CI 0.27–1.03, *p* = 0.13). In 3 RCTs, CRP showed a mean decrease of 43% from baseline at 7 days in the omega-3 group, compared to 20% in controls. In 2 RCTs, IL-10 had a mean increase of 47% in the omega-3 group compared to 18% in controls, suggesting an anti-inflammatory response.	Only one study reported a mild skin rash as an adverse event; otherwise, no adverse events were reported.
Jadhav et al., 2024 [185]	*RCT.* To investigate the comparative effect of omega-3 vs. octreotide infusion in AP within 48 h of onset.	Omega-3 1.5 g/kg daily intravenous infusion over 4 h for 7 consecutive days or until discharge. Octreotide dose not mentioned.	220	Significantly lower CRP levels in the omega-3 group by day 7 (50.2 ± 20.5 mg/L) compared to the octreotide group (75.4 ± 25.7 mg/L, *p* < 0.001). Significantly lower infection rate (7.3% vs. 15.5%, *p* = 0.03) and pancreatic necrosis (10.9% vs. 19.1%, *p* = 0.05) in the omega-3 group. Lower but not statistically significant organ failure rate (4.5% vs. 8.2%, *p* = 0.22) and mortality rate (2.7% vs. 6.4%, *p* = 0.18) in the omega-3 group.	None reported.

^1^ no data about adverse effects was mentioned in the study; RCT = randomized controlled trial; AP = acute pancreatitis; EN = enteral nutrition; PN = parenteral nutrition; SMC = standard medical care; CRP = C-reactive protein; IL-10 = interleukin-10; LoHS = length of hospital stay; ICU = intensive care unit.

#### 3.1.5. Anti-Ischemic Agents with Antioxidant Properties

1. Trimetazidine

Trimetazidine is a cytoprotective and anti-ischemic (anti-anginal) agent that inhibits fatty acid beta-oxidation, thus improving cellular glucose utilization as an advantageous alternative for energy production with less oxygen consumption [195]. By decreasing cellular ischemia, it combats intracellular acidosis, free radical production, and apoptosis. In addition to anti-ischemic properties, some studies demonstrated that trimetazidine also exerts antioxidant activity in patients with coronary heart disease and *cor pulmonale* by improving antioxidant levels, activating antioxidant enzymes, and indirectly decreasing the level of free radical oxidation products and lipid peroxides [196,197]. Also, trimetazidine was shown to modulate mitochondrial permeability following acute ischemia, by limiting calcium influx into mitochondria and thus preventing mitochondrial damage and cell apoptosis, a pivotal mechanism of AP pathogenesis [198].

Starting from these results, several authors studied the effects of trimetazidine on experimentally induced AP in rats (the duct injection model with Na-taurocholate). It was shown that trimetazidine administration (10 mg/kg/24 h via enteral route for 3 days) determined a significant reduction in tissue myeloperoxidase activity, MDA levels, as well as significant positive histological findings in terms of leukocyte infiltration, acinar cell necrosis, and apoptotic cell count (*p* < 0.05) [199], and also showed a positive effect on the mortality rate [200].

A study evaluating the effects of the intraperitoneal administration of trimetazidine in L-arginine-induced AP in rats (2 doses of 5 mg/kg 30 min and 12 h after induction) found significantly lower serum IL-1β, IL-6, and TNF-α levels and, after histopathological examination, significantly lower acinar cell necrosis, hemorrhage, perivascular inflammation, and edema in the treatment group [201]. Another study showed that the oral administration of trimetazidine at a dose of 20 mg/kg, 12 h after cerulein-induced AP in rats, significantly lowered serum IL-1, amylase, and lipase levels. TNF- α and white blood cell count were also lower in the treatment group, but the result was not statistically significant. The authors also described significantly decreased edema, inflammation, pancreatic fatty necrosis, and most importantly, significantly reduced apoptotic indices (2.05% in the treatment group vs. 3.57% in the placebo group) [202].

Trimetazidine has demonstrated clinical efficacy in various pathologies through its antioxidant and cytoprotective mechanisms, but its efficacy in the management of AP remains to be further evaluated and demonstrated. The results from preclinical models highlight an interesting field for research; nevertheless, the translational significance is still to be assessed. Future well-designed clinical trials are required to determine if trimetazidine’s anti-ischemic and anti-inflammatory properties can provide considerable benefits in the context of acute pancreatic inflammation.

2. Pentoxifylline

Pentoxifylline, also known as oxpentifylline, is a methylxanthine derivative extracted from cocoa beans, which exerts several antioxidant and anti-inflammatory activities, such as the maintenance of glutathione levels and mitochondrial viability, the inhibition of TNF-α synthesis, and the preservation of vascular endothelial functions [203]. Pentoxifylline was also proven to decrease the serum levels of C-reactive protein (CRP) and IL-6 in hemodialysis patients [204].

A small double-blind, randomized, placebo-controlled trial sought to determine the efficacy of pentoxifylline in patients with severe AP. There were no differences between the pentoxifylline group and placebo in terms of inflammatory markers, SIRS, and the APACHE II score; patients in the pentoxifylline group, however, had fewer intensive care unit (ICU) admissions and shorter ICU and hospital stays [205] (Table 5). The study further conducted a trial on a larger sample of AP patients using the same regimen of pentoxifylline and could not demonstrate any superiority over placebo. There were no significant differences in primary composite outcome (death, peri/pancreatic necrosis, persistent organ failure, persistent SIRS; *p* = 0.06), and this time, the pentoxifylline group was associated with longer hospital stays (*p* = 0.04) and higher readmission rates (*p* = 0.047) [206] (Table 5).

Another randomized controlled trial investigating the role of pentoxifylline in the prevention of post-endoscopic retrograde cholangiopancreatography (ERCP) AP showed that periprocedural supplementation did not protect against post-ERCP pancreatitis and did not prevent hyperamylasemia [207] (Table 5).

A case–control study evaluating the effects of pentoxifylline supplementation on clinical outcomes in AP showed that pentoxifylline did not decrease the severity of AP. However, the incidence of SIRS was significantly lower in the treatment group and, interestingly, patients who enrolled within 24 h of symptom onset experienced a greater reduction in pro-inflammatory markers and therefore tended to respond better to pentoxifylline than controls [208] (Table 5).

The therapeutic effectiveness of pentoxifylline in AP is still debated. Although its antioxidant and TNF-α inhibitory properties give a strong biological basis for therapeutic usage, clinical outcomes have varied across trials, ranging from small benefits to no meaningful impact. Importantly, the lack of significant adverse events reflects a favorable safety profile, which needs further examination. Future controlled randomized studies should be conducted to determine the appropriate patient group, dose regimens, and therapy timing in which pentoxifylline may provide clinical benefit in AP. 

Table 5 summarizes the main characteristics of studies investigating the effect of anti-ischemic agent supplementation in AP.

**Table 5:** Anti-ischemic agent supplementation in acute pancreatitis.

Antioxidant	First Author, Year	Study Design and Aim	Dose and Duration of Supplementation	No. of Participants	Outcomes	Adverse Effects
Trimetazidine		No human studies.			Significant anti-inflammatory effects, and significant impact on histopathologic changes in experimentally induced AP.	
Pentoxifylline	Kapetanos et al., 2007 [207]	*Randomized case–control.* To assess the efficacy of pentoxifylline in the prevention of post-ERCP AP.	400 mg orally the day before ERCP (2 and 10 pm) until the night after (6 am and 2 and 10 pm).	320	No impact on post-ERCP AP or hyperamylasemia.	Hemorrhage more prevalent in controls vs. treatment group (4.3% vs. 1.2%, respectively).
	Vege et al., 2015 [205]	*Double-blind placebo-controlled RCT.* To assess the efficacy of pentoxifylline in predicted severe AP.	400 mg t.i.d. for 72 h.	28	Fewer ICU admissions and shorter ICU and LoHS > 4 days (*p* = 0.03 and 0.046, respectively). No significant impact on serum inflammatory markers and SIRS or APACHE II score at day 3.	No adverse effects
	Wanichagool et al., 2019 [208]	*Case–control.* To evaluate the effects of pentoxifylline on clinical outcomes, the APACHE II score, and inflammatory markers in AP.	Administered for 72 h and within 48 h of AP onset.	54	No significant reduction in APACHE II score; (*p* = 0.27); significantly lower incidence of SIRS in treatment group (*p* = 0.048).	None reported ^1^
	Vege et al., 2020 [206]	*Double-blind placebo-controlled RCT.* To assess the efficacy of pentoxifylline on PCO (mortality, pancreatic necrosis, persistent OF, SIRS) in AP.	400 mg orally at enrollment followed by 400 mg t.i.d. for 72 h.	83	No significant impact on PCO (*p* = 0.06); pentoxifylline group associated with significantly longer LoHS (*p* = 0.04) and higher readmission rates (*p* = 0.047).	Not significant btw. the 2 groups.

^1^ no data about adverse effects was mentioned in the study; ERCP = endoscopic retrograde cholangiopancreatography; AP = acute pancreatitis; RCT = randomized controlled trial; ICU = intensive care unit; LoHS = length of hospital stay; SIRS = systemic inflammatory response syndrome; PCO = primary composite outcome; OF = organ failure.

### 3.2. Combined Antioxidant Therapy

A randomized double-blind placebo-controlled trial investigated the possible use and benefits of intravenous antioxidant therapy (selenium, ascorbic acid, N-ACC) in predicted severe AP. Surprisingly, patients who received antioxidant therapy had a longer length of hospital stay, a higher risk of developing multiple organ dysfunction compared to the placebo group, and a higher mortality rate at 7 days, despite an improved serum antioxidant status. Consequently, the study presented no evidence to support the use of antioxidants in patients with AP and organ dysfunction [171].

Furthermore, a prospective, randomized, placebo-controlled trial investigated the antioxidant therapy (ascorbic acid, N-ACC, Antoxyl forte—a combination of multiple antioxidants) associated with standard medical treatment vs. standard medical treatment alone in patients with early AP. Results showed a non-significant reduction in the lengths of hospital stay in the treatment group and a significant improvement in antioxidant status, but both groups developed similar local and systemic complications on days 1, 3, and 7 [209].

Another prospective randomized case–control study assessed the effect of vitamin supplementation (beta-carotene, ascorbic acid, alpha-tocopherol) on the outcomes of AP and the serum markers of oxidative stress at 7 days. Antioxidant therapy showed no impact on the incidence of organ dysfunction at day 7, and there was no significant difference between the complication rate for both groups at discharge/death. The mean length of hospital stay was lower in the antioxidant group, but the difference did not reach statistical significance. Additionally, antioxidant supplementation did not significantly reduce the markers of oxidative stress (defined by a raised level of MDA at admission) and neither restored the depleted levels of SOD and GSH, probably related to the low dose (1 g/day) of vitamin C used [210].

A 20-week double-blind placebo-controlled trial, including a total of 20 patients, 15 of whom had features of CP, aimed to find out if combined oral antioxidant therapy consisting of organic selenium, beta-carotene, ascorbic acid, alpha-tocopherol, and methionine can bring health benefits in patients with recurrent non-gallstone AP. During the study period, six patients in the control group had an AP flare, while no patient on active treatment experienced recurrent AP (*p* = 0.032). Moreover, active treatment had beneficial effects on pain, as was discovered from an analysis of visual analog pain scales and pain-score diaries [211]. However, a considerable number of patients had acute-on-chronic pancreatitis, and it is unclear if these results could be extrapolated to AP as well. After determining the serum biochemical profile of the examined patients, the authors found that selenium, beta-carotene, and alpha-tocopherol concentrations were significantly lower than in healthy controls and were normalized by antioxidant treatment. Moreover, exposure to antioxidants in the early phase of AP was associated with reduced oxidative stress through the preservation of the methionine trans-sulphuration pathway in pancreatic acinar cells [212].

A research study investigated the effect of combined antioxidant therapy (N-ACC + ascorbic acid + selenium) in severe AP and found no benefit regarding the survival rate after case–control analysis, even though ascorbic acid and selenium levels were restored to normal after supplementation in a subgroup of patients [170].

Other researchers performed a systematic review and meta-analysis of 22 RCTs on the effects of antioxidants on AP, CP, and post-ERCP AP. Combined antioxidant therapy (selenium, beta-carotene, L-methionine, ascorbic acid, and alpha-tocopherol), N-acetylcysteine, Gln, ascorbic acid, curcumin, SAMe, and allopurinol were used in the 22 included studies. While there was a positive effect on CP with the combination of selenium, beta-carotene, and SAMe, the results of the administration of other antioxidants in both AP and CP were heterogeneous, and no definite conclusions on their efficacy could be drawn. There was no association between the etiology of pancreatitis, the type of antioxidant therapy, and outcomes. The meta-analysis included four studies on allopurinol in post-ERCP AP. While antioxidant therapy failed to prevent post-ERCP AP in almost all clinical trials, the relative risk for the prevention of post-ERCP AP was non-significant for allopurinol vs. placebo administration [213].

A systematic review also investigated the role of antioxidant supplementation in the prevention of post-ERCP AP. Twelve RCTs involving 3110 patients since 1999 were included. Antioxidant therapy consisted of selenite, beta-carotene, and pentoxifylline (each one in a separate trial), N-ACC in three trials, and allopurinol in six trials, four of which were also included in the meta-analysis. Overall, antioxidant supplementation was associated with a non-significant reduction in the incidence of post-ERCP AP [8.6% vs. 9.7% in the control group; relative risk (RR) = 0.93; 95%CI: 0.82–1.06; *p* = 0.28].

After analyzing according to the type of antioxidant, the incidence of post-ERCP AP in patients receiving allopurinol was similar to that observed in patients receiving placebo (RR = 0.92; 95%CI: 0.78–1.08; *p* = 0.31). When stratifying according to dosage—low (300 and 400 mg), moderate (600 mg), and high (900 and 1200 mg)—there was still no statistically significant preventive effect of allopurinol on post-ERCP AP. The incidence of post-ERCP AP in trials using other antioxidants (selenite, beta-carotene, and pentoxifylline) was still not significantly lower than that of controls (8.9% vs. 9.7% in the control group; RR = 0.95; 95%CI: 0.77–1.18; *p* = 0.19). In conclusion, antioxidant supplementation showed no beneficial effect on the incidence of post-ERCP AP [214].

Despite the promising preclinical data, clinical evidence supporting the efficacy of combined antioxidant therapy in AP remains inconclusive. While some studies report improved antioxidant status, this has not consistently translated into better clinical outcomes, such as reduced organ failure, complications, or mortality. Results across trials remain heterogeneous, possibly due to variations in antioxidant combinations, dosing, timing, and patient selection. To better understand their potential, future research should identify which combinations, doses, and patient profiles are most likely to benefit from antioxidant treatment.

Table 6 summarizes the main characteristics of studies investigating the effect of combined antioxidant supplementation in AP.

**Table 6:** Combined antioxidant supplementation in acute pancreatitis.

Antioxidant	First Author, Year	Study Design and Aim	Dose and Duration of Supplementation	No. of Participants	Outcomes	Adverse Effects
Selenium (Se) + beta-carotene + ascorbic acid + alpha-tocopherol + methionine	Uden et al., 1990 [211]	*Double-blind, placebo-controlled RCT.* To assess the effect of antioxidants on recurrent AP and CP pain.	Daily oral total doses of Se 600 µg + beta-carotene 9000 IU + ascorbic acid 540 mg + alpha-tocopherol 270 IU + methionine 2 g for 20 weeks.	20	Significantly reduced incidence of recurrent AP (0 vs. 6 pts., *p* = 0.032); significant positive impact on chronic pancreatic pain compared to baseline (*p* < 0.001) and placebo (*p* = 0.049).	No adverse effects
Selenium + N-ACC + ascorbic acid + beta-carotene + alpha-tocopherol	Virlos et al., 2003 [170]	*Prospective case–control.* To assess the impact of antioxidants on outcomes in severe AP—antioxidant levels, morbidity and mortality, observed survival compared to predicted survival derived from logistic organ dysfunction score.	Day 1 and onwards: beta-carotene 9 mg + alpha-tocopherol 100 mg b.i.d. via NG tube. + IV continuous infusion. Day 1: N-ACC 300 mg/kg, Se 1000 µg, ascorbic acid 2 g. Day 2: N-ACC 150 mg/kg, Se 400 µg, ascorbic acid 2 g. Day 3 and onwards: N-ACC 75 mg/kg, Se 200 µg, ascorbic acid 1 g. Median duration of treatment: 18 (6–38) days.	46	Significant improvement in serum ascorbic acid (*p* = 0.003) and Se (*p* = 0.028); no significant impact on serum beta-carotene, alpha-tocopherol, and GSH; no benefit regarding survival.	No adverse effects
Selenium + ascorbic acid + N-ACC	Siriwardena et al., 2007 [171]	*Double-blind placebo-controlled RCT.* To assess the effect of antioxidants on clinical outcomes and antioxidant status in predicted severe AP (APACHE II score ≥ 8 on admission).	Se + N-ACC + ascorbic acid administered IV via continuous infusion. Day 1: N-ACC 300 mg/kg, Se 1000 µg, ascorbic acid 2 g. Day 2: N-ACC 150 mg/kg, Se 400 µg, ascorbic acid 2 g. Days 3–7: N-ACC 75 mg/kg, Se 200 µg, ascorbic acid 1 g.	43	No significant impact at 7 days on organ dysfunction (*p* = 0.33); higher MODS incidence at day 7 (*p* = 0.093); higher mortality rate (4 vs. 0), and longer LoHS vs. placebo (*p* = 0.34); significantly higher serum antioxidant levels (ascorbic acid and Se) and lower markers of oxidative stress (CRP and GSH) in the treatment group.	None directly attributable to antioxidant therapy
Ascorbic acid + N-ACC + Antoxyl forte—oral supplement containing vitamins (A, E, B_6_, C), Zn, Mg, Mn, Cu, Cr, Se, alpha lipoic acid, spirulina, lutein, zeaxanthin, L-cysteine, L-glutamic acid, and glycine	Sateesh et al., 2009 [209]	*Placebo-controlled RCT.* To investigate the impact of antioxidants on LoHS, complication rate, and biochemical markers of oxidative stress in early AP at days 1, 3, and 7.	Ascorbic acid 500 mg + N-ACC 200 mg every 8 h + Antoxyl forte 1 cps. Hourly for 7 days.	53	Non-significantly shorter LoHS in the treatment group vs. placebo (10.3 vs. 7.2 days, *p* = 0.07); similar complication rate; significantly reduced markers of oxidative stress in the treatment group (*p* = 0.03).	None reported ^1^
Ascorbic acid + alpha-tocopherol + beta-carotene	Bansal et al., 2011 [210]	*Prospective RCT with blinded endpoint assessment.* To assess the impact of vitamin supplementation on AP outcomes.	Ascorbic acid 1 g IV + alpha-tocopherol 200 mg orally + beta-carotene 10,000 IU IM b.i.d. for 14 days, followed by oral administration for all as soon as tolerated.	39	No impact on organ dysfunction/MODS at 7 days (*p* = 1.0 and 0.8, respectively); no significant impact on LoHS (*p* = 0.29); no significant impact on serum MDA, SOD, and GSH (*p* = 6.4, 0.74, and 0.68, respectively).	No adverse effects attributable to antioxidant therapy

^1^ no data about adverse effects was mentioned in the study; N-ACC = N-acetylcysteine; RCT = randomized controlled trial; AP = acute pancreatitis; CP = chronic pancreatitis; LoHS = length of hospital stay; NG = nasogastric; GSH = glutathione; MODS = multiple organ dysfunction syndrome; CRP = C-reactive protein; IM = intramuscular; MDA = malondialdehyde; SOD = superoxide dismutase.

## 4. Discussion

Oxidative stress and inflammatory signaling in AP are deeply implicated and interconnected in its pathogenesis. Following pancreatic injury, a dual pathological cascade is initiated. One pathway is characterized by the high production of reactive oxygen species (ROS), which directly negatively affect pancreatic acinar cells and impact mitochondrial function, leading to apoptosis or necrosis [215]. At the same time, redox-sensitive signaling pathways such as NF-κB, MAPKs, and PI3K/Akt activate the inflammatory axis, with high levels of pro-inflammatory cytokines (e.g., TNF-α, IL-1β, IL-6) and the recruitment of immune cells. All these processes induce local pancreatic damage and accelerate the progression to SIRS. Antioxidant therapy may disrupt this pathological cycle by neutralizing ROS and thus reducing the activation of the redox-sensitive inflammatory response. In patients with severe AP, clinical studies showed important oxidative stress, highlighted by elevated lipid peroxidation products such as malondialdehyde (MDA) and reduced levels of endogenous antioxidants such as vitamin C, β-carotene, and selenium [216].

Antioxidants can act at several points during the AP injury pathway. Vitamins C and E, melatonin, and polyphenols can work as direct free radical scavengers by preventing oxidative damage to DNA, proteins, and lipid membranes [217].

Other antioxidants can act indirectly through the modulation of endogenous defense systems. Curcumin and resveratrol activate the Nrf2–Keap1 pathway, an important regulator of cellular redox homeostasis. When Nrf2 is translocated into the nucleus, the up-regulation of the transcription of some antioxidant genes (glutathione peroxidase, superoxide dismutase, catalase, and heme oxygenase-1) is triggered, which enhances cellular resistance to oxidative injury [218]. Moreover, glutamine can restore glutathione in the liver and also protect the integrity of the gut barrier. Selenium is essential for glutathione peroxidase function [219].

Trimetazidine and pentoxifylline can also contribute to the improvement of mitochondrial activity and reduce pro-inflammatory cytokine synthesis, as well as improve pancreatic microcirculation [205,220].

Allopurinol blocks xanthine oxidase, a major generator of ROS during ischemia–reperfusion injury [221], by inhibiting enzymatic sources of ROS production. Some experimental agents (SkQ1) target mitochondria in order to prevent oxidative damage to the mitochondrial membrane [222].

**Figure 2:**
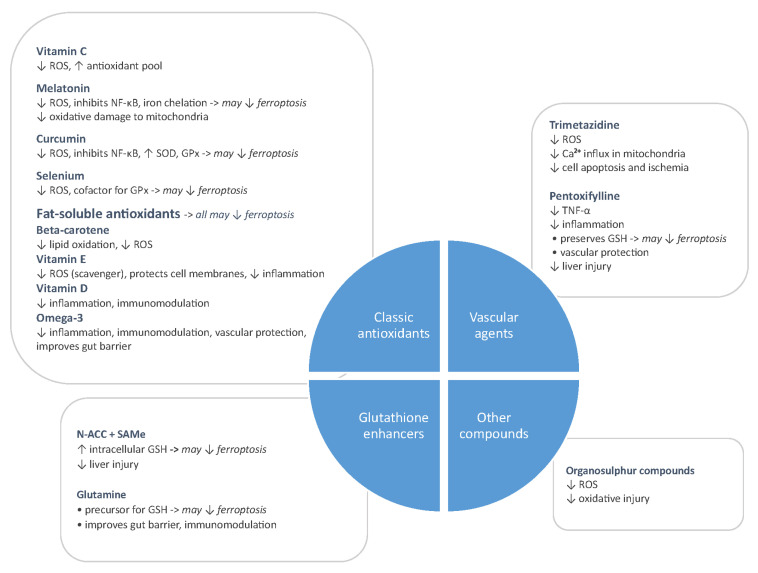
Overview of antioxidant compounds evaluated in experimental acute pancreatitis. ROS = reactive oxygen species; GPx = glutathione peroxidase; SOD = superoxide dismutase; N-ACC = N-acetylcysteine; SAMe = S-adenosyl methionine; TNF-α = tumor necrosis factor alpha; NF-κB = nuclear factor kappa B; GSH = glutathione.

Table 7 and Figure 2 summarize the main features of the antioxidants evaluated in clinical studies in AP.

AP, a disease which has virtually no specific treatment, has experienced multiple paradigm shifts recently—the shift from *nil per os* to early enteral feeding, the recent WATERFALL trial [223] changing the standard of early aggressive fluid resuscitation, as well as the development of highly advanced endoscopic procedures for the treatment of local complications, which have significantly reduced the need for surgery and thus improved survival and recovery rates. For more than a decade, antioxidant supplementation, as a more specific approach, was regarded as having the potential to significantly improve AP outcomes, but the results of clinical trials are far from being miraculous.

Despite restoring the antioxidant pool and/or decreasing the markers of oxidative stress, antioxidant supplementation failed to prove the expected results regarding the severity and mortality of AP; moreover, when used in combination or in higher doses, mortality even increased in some studies, raising the complicated question of whether oxidative stress should even be targeted in the acute phase of the disease, or if any compensatory mechanisms to restore homeostasis may be disrupted by high doses of antioxidants. Another issue could be a possible correlation between the systemic pro-oxidant status of the patient with AP, a status that can potentiate self-cure mechanisms (the liberation of glucocorticoids, pro-inflammatory cytokines, acute phase proteins, etc.). Following this hypothesis, antioxidants should not be administered in the early stage of this severe disease, but only after a period from onset, which might be determined by future research. Moreover, all studies so far have evaluated the endpoints of antioxidant supplementation during hospitalization, but the risk of post-AP mortality remains high, especially in the first 90 days after discharge [224], and the potential role of antioxidants in post-AP recovery should also be studied.

Some antioxidants with beneficial results in experimental settings did not show the same efficacy when translated to human studies, which may be a consequence of either inappropriate dosage, route of administration, and duration of therapy, or altered pharmacodynamics in vivo. One of the key pathological features of moderate-to-severe AP is microvascular dysfunction, with splanchnic vasoconstriction and compromised pancreatic perfusion. These circulatory alterations limit the efficient delivery of antioxidants to pancreatic tissue at the time of maximal injury. Additionally, systemic inflammation, increased capillary permeability, and fluid shifts further complicate pharmacokinetics and therapeutic targeting. Future clinical trials must focus on these aspects and consider the timing, route of administration, and hemodynamic context in which antioxidants are administered. A more precise understanding of the therapeutic window and microcirculatory dynamics in AP is essential for tailored therapeutic interventions.

## 5. Limitations

Despite its comprehensive aim, this review has several limitations in the current evidence on antioxidant supplementation in AP. Clinical studies show variability in study design, patient populations, antioxidant types, dosages, and timing of administration, making comparisons difficult. Most trials are small, focus on short-term outcomes, and lack standardized endpoints or consistent biomarkers. Preclinical data, although promising, are based on controlled animal models that do not fully reflect the heterogeneity and complexity of human AP. Additionally, the long-term effects and safety profile of antioxidant therapy remain underexplored, particularly in relation to post-AP recovery.

Unlike animal studies, where the model of experimental AP is homogeneous with respect to the study population and etiology (method of induced AP), clinical trials include cases of AP with heterogeneous etiopathogenesis (post-ERCP, alcohol- or drug-induced, hypertriglyceridemia, migrated gallstones, pancreatic cancer, etc.). In addition to different etiologies, the clinical status of patients at AP onset, the nutritional status, as well as the antioxidant pool and inflammation level vary significantly and make the study population even more heterogeneous. Furthermore, the unpredictable clinical course of AP, even among patients with initially mild symptoms, minimal organ dysfunction, and no major complications, adds an additional layer of complexity in evaluating therapeutic efficacy.

While experimental models have contributed to a better understanding of the oxidative and inflammatory mechanisms in AP, they cannot fully replicate the complexity of the human disease. Most preclinical models rely on chemically or hormonally (supramaximal cerulein stimulation, intraductal taurocholate infusion, or L-arginine overload) induced pancreatitis in healthy rodents, which lack human-like comorbidities (diabetes, obesity, or chronic alcohol consumption).

Moreover, clinical AP comes with a delay between symptom onset and the moment of diagnosis; thus, the therapeutic windows for interventions are short, and the therapy is often initiated after the peak of inflammatory and oxidative stress. These differences significantly limit the direct applicability of preclinical findings to patient care and underscore the need for improved translational models and refined clinical trial designs.

Promising agents in preclinical studies (e.g., resveratrol, trimetazidine, melatonin) were never used in humans or have not demonstrated consistent efficacy. The absence of reliable biomarkers to assess redox status further impairs personalized, targeted therapy, leading to empiric antioxidant supplementation.

These limitations highlight the necessity for multicenter randomized controlled trials with standardized protocols, outcome measures, and oxidative status characterization in order to establish the true therapeutic value of antioxidants in AP.

## 6. Conclusions

In a disease where specific pharmaceutical therapies are still limited, and supportive care remains the cornerstone of treatment, this review provides a detailed investigation into antioxidant supplementation as a therapeutic strategy in the treatment of AP.

Oxidative stress plays a key role in the pathophysiology of AP by enhancing acinar cell injury, inflammation, and systemic complications. Experimental studies have proved the efficacy of different antioxidant compounds (glutathione precursors, polyphenols, vitamins, trace elements, phytonutrients, omega-3 and fatty acids, anti-ischemic compounds, melatonin, etc.) to reduce oxidative injury, inflammatory markers, and ameliorate histological outcomes. However, the practical application of these findings to clinical benefit remains limited.

Although a multitude of animal studies demonstrated that antioxidant therapy has beneficial effects in experimental AP, human trials showed predominantly conflicting results, with some studies suggesting benefit while others showed no effect, or even potential harm, when antioxidants were administered in high doses or in combination.

The majority of clinical trials showed modest results, with no consistent evidence for improved survival, reduced organ dysfunction, or shorter hospital stays. Methodological differences between studies, delayed timing of intervention, and pharmacokinetic limitations can contribute to these discrepancies, while differences in routes of administration limit direct comparison and meta-analyses.

Therefore, while antioxidant-based interventions remain a promising adjuvant tool, current evidence does not support their real-life routine clinical use. Future studies should be centered on optimized dosing strategies, early administration protocols, targeted patient selection, and delivery methods of proper pharmaceutical forms.

## Figures and Tables

**Table 7:** An overview of the outcomes of the previously discussed antioxidants evaluated in clinical studies of acute pancreatitis.

Antioxidant (Type)	Proposed Mechanism of Action	Key Findings
**Vitamin E (α-tocopherol)**	Fat-soluble chain-breaking antioxidant; scavenges lipid peroxyl radicals and protects cell membranes.	Supplementation (400 IU/day) improved oxidative stress markers (↓ MDA, ↑ total antioxidant capacity). Clinical impact on AP severity not clearly demonstrated due to small sample.
**Vitamin C (ascorbic acid)**	Water-soluble antioxidant; cofactor for enzymatic reactions, regenerates other antioxidants, and may modulate immune function.	High-dose IV vitamin C (up to 10 g IV/day) showed improved antioxidant status and shorter hospital stay. Meta-analysis indicates reduced hospital stay but no clear impact on mortality or organ failure. Optimal dose/timing remains unclear; overall survival benefit not proven.
**β** **-carotene (provitamin A carotenoid)**	Lipid-soluble antioxidant; quenching of singlet oxygen and free radicals. Modulates immune responses and pancreatic β-cell function.	No reduction in overall post-ERCP AP incidence, but severe cases were fewer with β-carotene supplementation. AP patients have lower β-carotene levels, correlating with severity, suggesting a potential role in more severe cases.
**Glutamine (amino acid; immunonutrient)**	Precursor for glutathione; supports gut barrier and modulates immune response; indirect antioxidant.	Parenteral/enteral glutamine supplementation (0.3–0.5 g/kg) reduced infectious complications and mortality in several studies. Meta-analyses show significant reductions in hospital stay and multi-organ dysfunction with glutamine-enriched feeding. Now recommended as part of nutritional therapy in severe AP.
**N-acetylcysteine (N-ACC) (glutathione precursor)**	Restores intracellular glutathione; scavenges free radicals; inhibits NF-κB activation in acinar cells.	Despite strong rationale, clinical results are inconsistent. Prophylactic N-ACC did not significantly prevent post-ERCP AP in most trials. In AP, N-ACC improved biochemical markers (e.g., reduced MDA), but showed no confirmed benefit on clinical outcomes or length of hospital stay.
**S-adenosyl methionine (SAMe)**	Methyl donor that boosts trans-sulfuration to replenish glutathione; mitigates oxidative injury in the liver.	A 24 h infusion of SAMe+N-ACC showed no significant improvement in outcomes. The short duration and timing may have limited its efficacy; no clear evidence to support SAMe in AP at present.
**Selenium (trace element)**	Cofactor for glutathione peroxidase and other antioxidant enzymes; has anti-inflammatory and immune effects.	Selenium levels drop in severe AP, but supplementation trials yielded no significant clinical benefit. High-dose selenium did not reduce organ failure or mortality in severe AP. One study noted improved redox markers (↑GPx, ↓MDA), but without outcome improvements.
**Omega-3 fatty acids (e.g., fish oil)**	Anti-inflammatory and antioxidant effects via resolvins/protectins; reduces neutrophil ROS generation and modulates eicosanoids. Improves gut barrier function and immune regulation.	Supplementation with omega-3 IV lipid emulsion significantly lowered inflammatory markers and organ failure rate in AP. Meta-analysis of omega-3 in severe AP showed reduced infectious complications and a trend toward lower mortality. Patients receiving omega-3 had shorter ICU stays and improved immune status (higher IL-10) in some trials.
**Melatonin (endogenous hormone)**	Powerful free radical scavenger; inhibits NF-κB and inflammasome activation; stabilizes mitochondrial function.	Significantly reduced post-ERCP AP incidence. Endogenous melatonin levels correlate with milder AP; low serum melatonin is associated with severe AP. No therapeutic trial in established AP yet, but melatonin shows promise as adjunct prophylaxis.
**Curcumin (turmeric polyphenol)**	Multi-target antioxidant: scavenges ROS; inhibits NF-κB and MAPK signaling; up-regulates Nrf2/HO-1 pathway.	In chronic/tropical pancreatitis, curcumin (500 mg) lowered lipid peroxidation (MDA) and raised glutathione levels. In AP, nano-curcumin was associated with faster symptom resolution and shorter hospital stays, though larger studies are needed.
**Pentoxifylline (methylxanthine)**	Inhibits TNF-α and inflammatory cytokines; preserves glutathione and improves microcirculation.	Mixed results: initial small trial suggested fewer ICU admissions, but a larger RCT showed no improvement in organ failure or outcomes. No benefit in preventing post-ERCP AP.
**Combined antioxidant cocktails (multiple micronutrients)**	Various combinations aimed at broad antioxidant coverage (e.g., selenium + vitamin C + N-ACC; or vitamins A, C, E + selenium + methionine).	Mixed outcomes: In chronic/recurrent AP, long-term oral antioxidant therapy eliminated acute flares and improved pain over 20 weeks. However, in severe AP, early high-dose combination therapy failed to improve organ failure or mortality. Some trials even noted longer hospital stays with aggressive antioxidant use. The optimal combination, timing, and indication remain uncertain.

↑ = increase; ↓ = decrease; RCT = randomized controlled trial; AP = acute pancreatitis; ERCP = endoscopic retrograde cholangiopancreatography; ICU = intensive care unit; SIRS = systemic inflammatory response syndrome; MDA = malondialdehyde; GPx = glutathione peroxidase; N-ACC = N-acetylcysteine; IL = interleukin; TNF-α = tumor necrosis factor alpha; NF-κB = nuclear factor kappa B; MAPK = mitogen-activated protein kinase; ER stress = endoplasmic reticulum stress; Nrf2 = nuclear factor erythroid 2–related factor 2 (antioxidant response transcription factor).

## Abbreviations

The following abbreviations are used in this manuscript:

ALA	alpha-linolenic acid
AP	acute pancreatitis
ATP	adenosine triphosphate
BID	twice a day
CCT	controlled clinical trial
CG	controlled group
CP	chronic pancreatitis
CRP	C reactive protein
DAMPs	damage-associated molecular patterns
DHA	docosahexaenoic acid
DNA	deoxyribonucleic acid
EEN	early enteral nutrition
EN	enteral nutrition
EPA	eicosapentaenoic acid
ER stress	endoplasmic reticulum stress
ERCP	endoscopic retrograde cholangiopancreatography
ESPEN	European Society for Parenteral and Enteral Nutrition
GPx	glutathione peroxidase
GSH	reduced glutathione
HS-CRP	High-Sensitivity C reactive protein
ICU	intensive care unit
IL	interleukin
IM	intramuscular
IR	intrarectal
IU	international units
LoHS	length of hospital stay
LPS	lipopolysaccharide
MAPK	Mitogen-Activated Protein Kinase
MDA	malondialdehyde
MOSF	Multiple Organ Dysfunction Syndrome
MPO	myeloperoxidase
N-ACC	N-acetylcysteine
NF-κB	Nuclear Factor κB
NG	naso-gastric
NO	nitric oxide
Nrf2	Nuclear factor erythroid 2-related factor 2
OF	organ failure
PCO	primary composite outcome
PN	parenteral nutrition
PUFAs	polyunsaturated fatty acids
RCT	randomized controlled trials
ROS	reactive oxygen species
SAMe	S-adenosyl methionine
SAP	severe acute pancreatitis
SARS-CoV2	severe acute respiratory syndrome coronavirus 2
SIRS	systemic inflammatory response syndrome
SMC	standard medical care
SOD	superoxide dismutase
TNF	tumor necrosis factor
TPN	total parenteral nutrition
WON	walled-off necrosis
